# Novel plasmid-free *Gluconobacter oxydans* strains for production of the natural sweetener 5-ketofructose

**DOI:** 10.1186/s12934-020-01310-7

**Published:** 2020-03-04

**Authors:** Svenja Battling, Karen Wohlers, Chika Igwe, Angela Kranz, Matthias Pesch, Astrid Wirtz, Meike Baumgart, Jochen Büchs, Michael Bott

**Affiliations:** 1 AVT-Biochemical Engineering, RWTH Aachen University, Forckenbeckstraße 51, 52074 Aachen, Germany; 2 IBG-1: Biotechnology, Institute of Bio- and Geosciences, Forschungszentrum Jülich, 52425 Jülich, Germany

**Keywords:** *Gluconobacter oxydans*, Sweetener, Chromosomal integration, 5-ketofructose, Fructose dehydrogenase

## Abstract

**Background:**

5-Ketofructose (5-KF) has recently been identified as a promising non-nutritive natural sweetener. *Gluconobacter oxydans* strains have been developed that allow efficient production of 5-KF from fructose by plasmid-based expression of the fructose dehydrogenase genes *fdhSCL* of *Gluconobacter japonicus*. As plasmid-free strains are preferred for industrial production of food additives, we aimed at the construction of efficient 5-KF production strains with the *fdhSCL* genes chromosomally integrated.

**Results:**

For plasmid-free 5-KF production, we selected four sites in the genome of *G.* *oxydans* IK003.1 and inserted the *fdhSCL* genes under control of the strong P264 promoter into each of these sites. All four recombinant strains expressed *fdhSCL* and oxidized fructose to 5-KF, but site-specific differences were observed suggesting that the genomic vicinity influenced gene expression. For further improvement, a second copy of the *fdhSCL* genes under control of P264 was inserted into the second-best insertion site to obtain strain IK003.1::*fdhSCL*^*2*^. The 5-KF production rate and the 5-KF yield obtained with this double-integration strain were considerably higher than for the single integration strains and approached the values of IK003.1 with plasmid-based *fdhSCL* expression.

**Conclusion:**

We identified four sites in the genome of *G.* *oxydans* suitable for expression of heterologous genes and constructed a strain with two genomic copies of the *fdhSCL* genes enabling efficient plasmid-free 5-KF production. This strain will serve as basis for further metabolic engineering strategies aiming at the use of alternative carbon sources for 5-KF production and for bioprocess optimization.

## Background

The strictly aerobic acetic acid bacterium *Gluconobacter oxydans* contains at least eight membrane-bound dehydrogenases catalyzing the rapid chemo-, regio-, and stereoselective oxidation of sugars, alcohols, and polyols in the periplasm to organic acids, aldehydes, and ketones [1]. The resulting electrons are transferred via ubiquinone to the terminal cytochrome *bo*_3_ oxidase [2] or a cyanide-insensitive *bd*-type oxidase [3], both of which reduce oxygen to water. Due to the distinctive properties of the membrane-bound dehydrogenases, *G.* *oxydans* has become an important workhorse for oxidative biotransformations in biotechnology [4–8]. The first industrial application of *G.* *oxydans* was in vitamin C production via the Reichstein-Grüssner process [9], where the sorbitol dehydrogenase catalyzes the oxidation of D-sorbitol to L-sorbose [10]. Other products whose synthesis involves *G.* *oxydans* include dihydroxyacetone [11] or the anti-diabetic drug miglitol [12, 13].

Due to the vigorous incomplete periplasmic substrate oxidation of *G.* *oxydans,* only a small fraction of the carbon source is taken up into the cytoplasm and enters the central carbon metabolism, which is characterized by an incomplete glycolysis and an incomplete tricarboxylic acid (TCA) cycle [14]. Glucose is metabolized predominantly via the oxidative pentose phosphate pathway and to some extent via the Entner–Doudoroff pathway [15]. Pyruvate is partly converted to acetate as final product by pyruvate decarboxylase and acetaldehyde dehydrogenase [16]. As a consequence of the small fraction of substrate metabolized within the cell, the biomass yield of *G.* *oxydans* is quite low (about 0.1 g cell dry weight/g glucose). As this causes increased costs for biomass synthesis, metabolic engineering was used to create *G.* *oxydans* strains with improved biomass yield on glucose by reducing or avoiding its oxidation to gluconate [16, 17]. One of the resulting strains, IK003.1, which is derived from the parent strain 621H, lacks both the membrane-bound and the soluble glucose dehydrogenase as well as pyruvate decarboxylase. It has a 60% increased biomass yield on glucose and accumulates pyruvate instead of acetate [17].

Excess sugar consumption is associated with obesity and various diseases such as cardiovascular diseases or type II diabetes [18–20]. Thus, the food industry aims at the replacement of nutritive sugars such as sucrose or high-fructose corn syrup by non-nutritive sugar substitutes such as sucralose [21]. Various artificial and natural sweeteners are already available on the market, but most of them have drawbacks. Polyols such as d-sorbitol or d-xylitol show laxative effects [18], whereas compounds such as acesulfame K or steviol glycosides have a bitter off-taste [22]. 5-Keto-d-fructose (5-KF) is considered as a potential non-nutritive natural sweetener [23], which has been found e.g. in white wine [24, 25]. It shows a sweet taste quality identical to that of fructose and has a similar intrinsic sweet threshold concentration of 16.4 mmol/L [26]. Therefore, the development of processes for the production of 5-KF has recently gained attention.

*G.* *oxydans* is able to oxidize fructose to 5-KF when cultivated with fructose [25] or with mannitol, which is initially oxidized to fructose [27]. However, the enzyme responsible for 5-KF formation in *G.* *oxydans* is not known yet. In contrast, fructose dehydrogenase (FDH), a membrane-bound enzyme catalyzing the periplasmic oxidation of fructose to 5-KF with ubiquinone as electron acceptor, has been isolated and characterized from *Gluconobacter* *japonicus* NBRC3260 (formerly *Gluconobacter* *industrius* IFO3260) [28, 29]. It is a heterotrimeric enzyme composed of a small subunit with a putative Tat signal peptide (FdhS), a Sec-secreted cytochrome *c* subunit with three CXXCH motifs for covalent heme attachment and a C-terminal transmembrane helix anchoring the entire complex to the membrane (FdhC), and a large subunit with a covalently bound flavin adenine dinucleotide (FAD) cofactor (FdhL). It lacks a signal peptide and is presumably exported via the Tat system in complex with FdhS. The corresponding genes are organized in the polycistronic *fdhSCL* transcription unit [29]. Plasmid-based expression of the *G.* *japonicus fdhSCL* genes under the control of an *adhAB* promoter in a Δ*adhA* mutant of *G.* *oxydans* NBRC12528 lead to 20-fold higher FDH activity compared to *G.* *japonicus* wild-type cells, confirming the successful synthesis of FDH in this heterologous host [29].

The application of FDH for the targeted production of 5-KF was reported only recently [30]. A strain of *G.* *oxydans* 621H expressing the *fdhSCL* genes of *G. japonicus* under the control of the strong constitutive promoter P264 on the broad host range plasmid pBBR1p264-*fdhSCL*-ST showed good growth on fructose and formed 5-KF with a yield of 89 mol% 5-KF per fructose. Moreover, when combined with a second *G.* *oxydans* strain secreting the sucrase SacC of *Zymomonas mobilis*, this bacterial community was able to convert either purified sucrose or sucrose present in sugar beet extract to glucose and fructose and oxidize the latter to 5-KF with molar yields of > 90% and > 80%, respectively [30]. Most recently, 5-KF production from sucrose was reported for a *G. oxydans* strain with a chromosomal *fdhSCL* integration and plasmid-based expression of an invertase, resulting in conversion of 84 ± 2 mol% of the fructose units of sucrose into 5-KF [31]. *G.* *oxydans* carrying the expression plasmid pBBR1p264-*fdhSCL*-ST was also used for bioprocess development. When cultivated in a 2 L bioreactor with constant fructose feeding, 5-KF up to 489 g/L, yields up to 0.98 g 5-KF/g fructose and space-time yields up to 8.2 g/L/h were reached, demonstrating the efficiency of this 5-KF production process [26].

Considering an industrial implementation, 5-KF production with plasmid-free strains would be desirable. Therefore, the current study aimed at the development and characterization of *G.* *oxydans* strains with genomically integrated *fdhSCL* genes. Initially, four strains of *G.* *oxydans* IK003.1, each harboring a single copy of the *fdhSCL* genes at different genomic loci, were constructed and analyzed regarding 5-KF production. Based on these results, a strain carrying two genomic *fdhSCL* copies under control of the P264 promoter was constructed, which showed even better growth on fructose than the single-copy strains and formed 5-KF with yields of up to 0.82 g/g.

## Results and discussion

### Selection of genomic integration sites for the *fdhSCL* genes and design of integration constructs

In this study, we wanted to generate plasmid-free *G.* *oxydans* strains for the production of 5-KF by genomic integration of the FDH genes *fdhSCL* from *G. japonicus*. Because the genomic vicinity can influence the expression of integrated genes, we selected four different integration sites in the genome of *G.* *oxydans* IK003.1. For one of the insertions, the genes GOX2096-GOX2095 (GOX_RS11750) for a sorbitol dehydrogenase large subunit were replaced by the *fdhSCL* genes. GOX2096-GOX2095 represent an authentic frameshift in *G.* *oxydans* 621H leading to an inactive enzyme [14, 32]. Therefore, the replacement does not lead to a metabolic deficiency. In the case of the other three integration sites, the *fdhSCL* genes were inserted into intergenic regions (IGRs) without deleting any part of the genome. The IGRs were chosen based on the following criteria: (i) the genomic positions should be close to the origin of replication to profit from a positive gene dosage effect; (ii) the positions should be located between the 3′-ends of two convergent genes to avoid an interference with the regulation of neighboring genes; (iii) the expression levels of the adjacent genes should vary for the three sites to test the influence of the genomic vicinity on *fdhSCL* expression; (iv) the chromosomal gene upstream of the *fdhSCL* genes should possess a terminator to enable comparison of the three loci without readthrough from an upstream promoter. Applying those criteria and using RNAseq data [33] for the evaluation of the expression levels of the neighbouring genes, the following positions were selected: igr1 between GOX0013 and GOX0014, igr2 between GOX0028 and GOX0029, and igr3 between GOX0038 and GOX0039. The exact integration sites were positioned directly downstream of the terminator of the upstream gene, as predicted with the online tool ARNold [34].

**Fig. 1:**
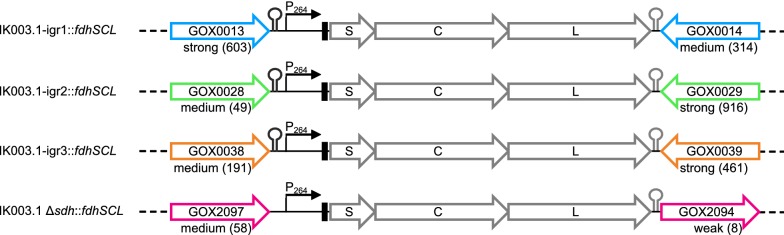
Scheme of the loci used for targeted integration of the *fdhSCL* genes into the genome of *G.* *oxydans* IK003.1. Shown are the integration sites with the flanking genes, the P264 promoter (black arrow), a consensus RBS (black rectangle), the *fdhSCL* genes (gray arrows), and terminators (black hairpin = native, gray hairpin = inserted). The transcription strength of the flanking genes (strong, medium, weak) and the normalized read count (given by Fragments Per Kilobase of transcript per Million mapped reads (FPKM) values in brackets) are also indicated. FPKM values were determined via RNAseq with *G.* *oxydans* 621H grown on mannitol (exponential growth phase) [33]. To differentiate the transcription levels, the FPKM values (range of 0–446,627) were grouped in the lowest 25% (0–38, weak), the median 50% (38–318, medium) and the highest 25% (318–446,627, strong)

The *fdhSCL* integration fragments were composed of 500 bp upstream DNA of the respective insertion site followed by the strong constitutive P264 promotor, the consensus ribosome binding site (RBS) AGGAG [33], the *fdhSCL* genes with an ATG start codon instead of TTG for *fdhS*, which was shown to be beneficial for 5-KF production [29], the bidirectional 100 bp terminator region downstream of GOX0028, which led to efficient termination according to RNA seq data [33], and 500 bp downstream DNA of the respective insertion site. The integration fragments were inserted into the suicide vector pAJ63a [35] and transferred into *G.* *oxydans* IK003.1 by conjugation, followed by a two-step homologous recombination protocol as outlined in the Methods section. The resulting integration strains were checked via colony-PCR and named IK003.1-igr1::*fdhSCL*, IK003.1-igr2::*fdhSCL*, IK003.1-igr3::*fdhSCL*, and IK003.1 Δ*sdh*::*fdhSCL* (Fig. 1).

### Shake flask cultivations with different *G. oxydans* IK003.1::*fdhSCL* strains with 18 g/L and 80 g/L fructose

**Fig. 2:**
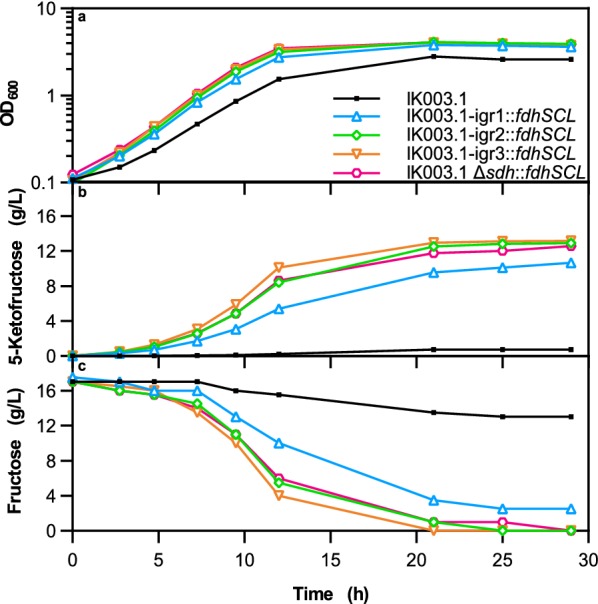
Growth, 5-KF formation, and fructose consumption of the indicated *G.* *oxydans* strains in shake flasks. Depicted is **a** the cell density as OD_600_, **b** the 5-KF concentration, and **c** the fructose concentration determined by HPLC (method A). The strains were cultivated in 100 mL complex medium with 18 g/L fructose in 500 mL baffled shake flasks at 30 °C, 130 rpm, a shaking diameter of 50 mm, and 85% humidity. Shown are mean values of biological duplicates

Shake flask cultivations with offline sampling, using the four integration strains as well as IK003.1 as reference, were performed to assess whether the integration strains produce more 5-KF than the parental strain. Cultivations were performed with 18 g/L fructose (= 100 mM). Growth, fructose consumption, and 5-KF formation are shown in Fig. 2. After 29 h of cultivation, when no further growth was observed, IK003.1-igr3::*fdhSCL,* IK003.1-igr2::*fdhSCL*, and IK003.1 Δ*sdh*::*fdhSCL* had completely consumed the fructose, resulting in higher 5-KF concentrations than observed for IK003.1-igr1::*fdhSCL* with 2.5 g/L residual fructose. The integration strains showed 15–22% higher growth rates (0.31 h^−1^ –0.33 h^−1^) than the reference strain IK003.1 (0.27 h^−1^) and reached higher final cell densities. Due to the lack of the *fdhSCL* genes, IK003.1 cannot generate energy by periplasmic fructose oxidation, which explains slower growth and lower biomass formation. Nevertheless, the reference strain produced small amounts of 5-KF, reaching a yield of 0.04 g/g. Low 5-KF production has also previously been described for *G.* *oxydans* grown on mannitol [27]. Here fructose oxidation to 5-KF was presumably catalyzed by the membrane-bound polyol dehydrogenase mSldAB, since a side activity for fructose oxidation has been described for a *Gluconobacter thailandicus* mSldAB homolog [36]. All integration strains formed 5-KF, but the kinetics differed (Fig. 2b). Strain IK003.1-igr3::*fdhSCL* showed the fastest 5-KF production, strains IK003.1-igr2::*fdhSCL* and IK003.1 Δ*sdh*::*fdhSCL* were somewhat slower, and IK003.1-igr1::*fdhSCL* was much slower than the other strains. The 5-KF yields after 29 h were comparable for the three fastest strains (0.76 g/g for IK003.1-igr3::*fdhSCL* and IK003.1-igr2::*fdhSCL*, 0.74 g/g for IK003.1 Δ*sdh*::*fdhSCL*), but lower for IK003.1-igr1::*fdhSCL* (0.61 g/g). This experiment already showed an influence of the integration site on 5-KF production with the ranking IK003.1-igr3::*fdhSCL* > IK003.1-igr2::*fdhSCL* > IK003.1 Δ*sdh*::*fdhSCL* > IK003.1-igr1::*fdhSCL*. This ranking was later confirmed in additional experiments.

To further investigate the four different integration sites, cultivations with online monitoring of respiratory activity in shake flasks were performed using the Respiration Activity MOnitoring System (RAMOS). Measuring the oxygen transfer rate (OTR) and the carbon dioxide transfer rate (CTR) provides extremely useful information about the metabolic activities and the physiological state of the microorganisms [37, 38]. 5-KF production from fructose is an oxidation reaction catalyzed by FDH and the electrons are transferred in the respiratory chain to oxygen as the final electron acceptor [26, 39]. Hence, 5-KF formation contributes to the OTR kinetics in addition to other catabolic activities of the *G. oxydans* strains. Cultivations with online monitoring of the respiratory activity were performed to obtain additional information on the growth behavior of the four integration strains using IK003.1 and the plasmid-containing strain IK003.1 pBBR1p264-*fdhSCL*-ST as reference (Additional file 1: Fig. S1). Increasing the fructose concentration from 18 g/L to 80 g/L led to a decrease of the 5-KF yield for all four integration strains, reaching values between 0.36 g/g and 0.58 g/g (Additional file 1: Table S1). Residual fructose concentrations of 25 g/L to 45 g/L were detected for the integration strains (Additional file 1: Table S1). IK003.1 pBBR1p264-*fdhSCL*-ST reached a much higher yield of 0.88 g/g.

The kinetics of OTR, CTR, and the respiratory quotient (RQ = CTR/OTR) observed for the six strains (Additional file 1: Fig. S1a–c) are likely caused by differences in the fructose oxidation rate, as a consequence of varying *fdhSCL* expression levels, which will be discussed below. The plasmid-containing strain IK003.1 pBBR1p264-*fdhSCL*-ST presumably has the highest FDH activity and showed the highest OTR. The maximal total oxygen consumption (TOC) of 220 mmol/L was reached already after 19 h (Additional file 1: Fig. S1d). Due to the rapid oxidation of fructose to 5-KF, less fructose is available for uptake and intracellular oxidation, resulting in the lowest total carbon dioxide evolution (TCE) of 50 mmol/L for IK003.1 pBBR1p264-*fdhSCL*-ST (Additional file 1: Fig. S1e). The reference strain IK003.1 had only a very low activity for fructose conversion to 5-KF and a very low maximal TOC of 80 mmol/L. The CTR was also quite low for IK003.1, because the strain may be energy-limited in the absence of FDH-based respiration, resulting in a maximal TCE of only 75 mmol/L. The 5-KF production of the four integration strains was slower than in the plasmid-based strain, resulting in a lower OTR. A maximal TOC of 250 mmol/L was reached for the integration strains at the end of the cultivation, except for IK003.1-igr1::*fdhSCL*. At the same time, more fructose was taken up and catabolized within the cell, resulting in higher CTR. The maximal TCE is two to four times higher for all integration strains in comparison with the plasmid-containing strain (Additional file 1: Fig. S1e). The kinetics of the RQ reflects these differences (Additional file 1: Fig. S1c).

Another parameter that probably influences the kinetics of OTR and CTR is the pH value. Strain IK003.1 forms pyruvate (pK_a_ 2.49) instead of acetate (pK_a_ 4.78), leading to a stronger acidification of the medium. In the experiment shown in Additional file 1: Fig. S1, pyruvate formation is dependent on the rate of intracellular fructose catabolism and thus should be reflected by the CTR value. Consequently, the integration strains should show a stronger acidification than the plasmid-containing strain and IK003.1, which was confirmed by the pH values measured after 29 h of cultivation (Additional file 1: Table S1). The pH influences the FDH activity and thus fructose consumption. The pH optimum for FDH is around 4 and activity decreases slightly at higher pH values, but strongly at lower pH, with no activity observed at pH 3 [29]. It is therefore likely that the OTR and CTR kinetics observed for the integration strains with a slow decrease after the maximum (Additional file 1: Fig. S1) and the incomplete fructose consumption (Additional file 1: Table S1) is caused by inhibition of FDH and other enzymes, such as those of the pentose phosphate pathway [40], due to acidification.

### Influence of buffering on growth and 5-KF production during cultivation of IK003.1-igr1::*fdhSCL* with 80 g/L fructose in a RAMOS device

The decreasing pH during the cultivation had presumably a negative influence on growth and product formation during the cultivation. Therefore, it was investigated whether pH control has a positive effect on growth and product formation. CaCO_3_ is sometimes used in shake flask cultivations for buffering, but it has several disadvantages including turbidity of the medium [41]. Therefore, 2-(*N*-morpholino)ethansulfonic acid (MES) buffer with a pK_a_ value of 6.1 was used [42]. Strain IK003.1-igr1::*fdhSCL* was chosen to compare cultivations without buffer and with 50 mM, 100 mM, and 150 mM MES buffer.

**Fig. 3:**
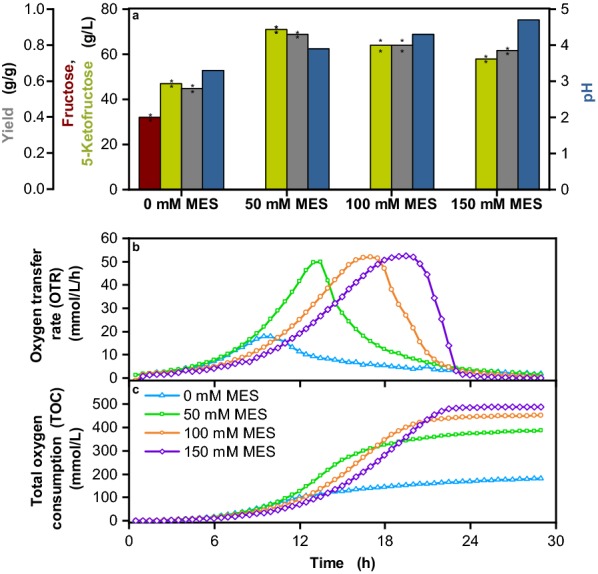
Cultivation of *G.* *oxydans* IK003.1-igr1::*fdhSCL* in a RAMOS device with 80 g/L fructose and different MES buffer concentrations. Depicted is **a** the residual fructose concentration (red), the 5-KF concentration (green), the yield g/g (grey), and the pH (blue), **b** the oxygen transfer rate (OTR) and **c** the total oxygen consumption (TOC). Cultivations were performed at 30 °C, 350 rpm, V_L_ = 10 mL in 250 mL flasks, pH_start_ = 6 and a shaking diameter of 50 mm in complex medium with 80 g/L fructose and MES buffer at concentrations of 0 mM, 50 mM, 100 mM and 150 mM. Shown are mean values of duplicates and the concentrations determined for fructose and 5-KF in the two experiments as well as the resulting yields are shown as stars

The addition of MES buffer had a strongly positive effect on 5-KF formation (Fig. 3). As expected, the pH value at the end of the cultivation increased from 3.3 for unbuffered cultivation to 4–4.7 for the cultivations with MES buffer (Fig. 3a). Fructose was completely consumed in the buffered cultures, resulting in a significantly increased 5-KF formation of up to 71 g/L and an increase of the yield from 0.56 g/g for the unbuffered culture to 0.86 g/g for the culture with 50 mM MES (Fig. 3a). At 100 mM and 150 mM MES, the 5-KF concentration and the yield were lower than at 50 mM MES, but the final OD_600_ was higher (4.1 for 0 mM MES, 4.9 for 50 mM MES, 5.4 for 100 mM MES, 5.9 for 150 mM MES). Higher pH values thus favor biomass formation and decrease 5-KF production.

The OTR kinetics of cultivations with the different MES concentrations showed clear differences (Fig. 3b). The maximal OTR of all MES-buffered cultures was about 50 mmol/L/h and thus 2.5-fold higher than for the unbuffered culture. At 100 mM and 150 mM MES, the OTR slowed down compared to 50 mM MES. This is most likely due to the higher osmolality of the medium, which increased from 0.56 Osmol/kg for unbuffered medium to 0.78 Osmol/kg for medium with 150 mM MES. The negative influence of osmolality on growth of *G.* *oxydans* has already been described [41, 43].

The kinetics of TOC is shown in Fig. 3c. As expected from the OTR kinetics, the TOC values of the MES-containing cultures were much higher than that of the unbuffered culture. Increasing MES concentrations correlated with increased TOC values, which can be explained by the fact that higher MES concentration led to higher biomass formation and thus a higher fraction of fructose was metabolized intracellularly, leading to more reducing equivalents per fructose than its simple oxidation to 5-KF.

Despite the increased lag phase, the positive effect of MES buffer regarding pH, TOC, OD_600_, and yield predominates. Therefore, subsequent shake flasks experiments were conducted with 150 mM MES buffer, which led to the highest TOC and the highest pH at the end of the cultivation. This was important for further experiments with fructose concentrations up to 210 g/L performed in shake flasks (Additional file 1: Fig. S5, Tab. S2).

### Comparison of different *G. oxydans* strains during cultivation in a RAMOS device with 80 g/L fructose and 150 mM MES buffer

**Fig. 4:**
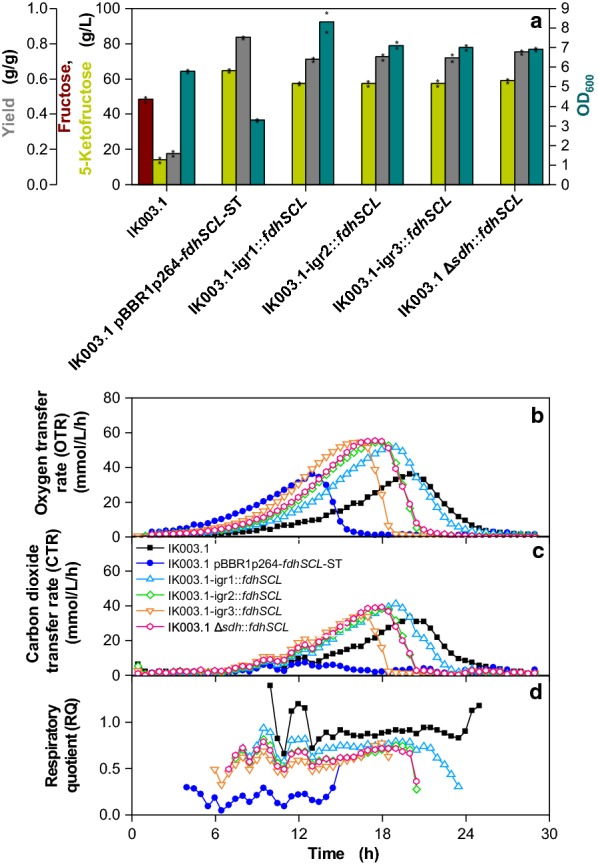
Cultivation of the indicated *G.* *oxydans* strains in a RAMOS device with 80 g/L fructose and 150 mM MES. Depicted is **a** the residual fructose concentration and the 5-KF concentration (determined by HPLC method B), the yield g/g, and the optical density (OD_600_) after 29 h, **b** the oxygen transfer rate (OTR), **c** the carbon dioxide transfer rate (CTR), and **d** the respiratory quotient (RQ, shown for OTR values above 5 mmol/L/h). The strains were cultivated in complex medium with 80 g/L fructose and 150 mM MES at 30 °C, 350 rpm, V_L_ = 10 mL in 250 mL flasks, pH_start_ = 6 and a shaking diameter of 50 mm. Shown are mean values of duplicates and the values determined for optical density, the concentrations of fructose and 5-KF, and the resulting yields in the two experiments are shown as stars

Based on the positive effect of MES buffering on 5-KF production by IK003.1-igr1::*fdhSCL*, these conditions were used to compare the four different integration strains among each other, with the parent strain IK003.1, and with the plasmid-containing strain IK003.1 pBBR1p264-*fdhSCL*-ST (Fig. 4).

As expected from the previous experiments, all strains except for IK003.1 consumed fructose completely and the final pH values varied between 4.3 and 4.6. 5-KF formation by the four integration strains was very similar (57–59 g/L), corresponding to yields of 0.71–0.75 g/g (Fig. 4a). Compared to the unbuffered cultures (Additional file 1: Table S1) this was an increase of 1.2- to 2-fold. The plasmid-containing strain formed 65 g/L 5-KF corresponding to a yield of 0.84 g/g.

The final OD_600_ of the integration strains ranged between 6.9 and 8.3 (Fig. 4a). In view of the comparable 5-KF formation of the four strains, these differences were unexpected and apparently are a consequence of the different genomic integration sites. The plasmid-containing strain had a much lower final OD_600_ of only 3.3 compared to the integration strains. As discussed above, this is caused by the higher copy number resulting in more *fdhSCL* transcript, resulting in faster fructose oxidation and a lower amount of fructose entering the cell. Additionally, a longer period of fructose oxidation by FDH also provides a longer period of energy supply for biomass formation from components of the yeast extract. Once fructose has been exhausted, growth is no longer sustained.

The statements made above are supported by the OTR and CTR kinetics (Fig. 4b, c). The plasmid-based strain reached its OTR maximum of 36 mmol/L/h already after 13 h, whereas the integration strains reached their maximum of ~ 55 mmol/L/h after 17 h –19 h (Fig. 4b). CTR was very low for the plasmid-based strain and much higher for the integration strains. This is in line with a higher intracellular fructose catabolism and a presumably higher consumption of yeast extract components (Fig. 4c). For strain IK003.1, both OTR and CTR were retarded compared to the integration strains. These kinetics are due to slow intracellular oxidation of fructose. In line with these data, the RQ value for IK003.1 was the highest of all strains (about 0.9), whereas the RQ of the plasmid-based strain was very low with a value of about 0.2. The integration strains showed RQ values in the range of 0.6–0.8 (Fig. 4d).

This experiment revealed that when using buffered conditions, the *fdhSCL* integration strains approached the 5-KF titer and the yield of the strain with plasmid-based *fdhSCL* expression. With respect to the four genomic integration sites, the differences in 5-KF titer and yield were rather small, but IK003.1-igr3::*fdhSCL* showed the fastest growth followed by IK003.1 Δ*sdh*::*fdhSCL* and IK003.1-igr2::*fdhSCL*.

### Scale up and fermentation in 2 L fermenter with IK003.1-igr3::*fdhSCL*

An important step for the industrial implementation of 5-KF production with a plasmid-free strain is the design and establishment of a suitable fermentation process. As a first step, a scale up from shake flasks to a 2 L fermenter was performed with strain IK003.1-igr3::*fdhSCL*, which was the best performing integration strain so far. To allow a direct comparison between shake flask (RAMOS system) and fermenter, the same medium, initial pH, and temperature were used for both cultivation devices. The shake flask was filled with inoculated medium by sterile transfer of a sample of the fermenter culture to ensure optimal comparability. In shake flask cultivations with 80 g/L fructose, no oxygen limitation was detected in the RAMOS devices. Hence, for a successful scale-up, oxygen limitation in the fermenter had to be avoided. For that reason, the dissolved oxygen tension (DOT) was kept above 30% by adjusting the agitation speed, whereas the aeration rate in the fermenter was kept constant at 1 vessel volume per minute (vvm). To verify the successful scale-up, online data (OTR, CTR, RQ) and offline data (OD_600_, pH, fructose and 5-KF concentration) were measured and compared. The results are shown in Additional file 1: Fig. S2. No significant differences in both, online and offline data, were found. Additional fermentations were performed as first optimization steps and confirmed the consistency of online and offline data (Additional file 1: Fig. S3 and Fig. S4). Hence, the scale up to a 2 L fermenter was successful and the process could be further optimized.

Higher product concentrations are desirable for an industrial application. To achieve higher 5-KF titers, the fructose concentration in batch fermentations was increased. To identify the optimal initial fructose concentration for the cultivation of IK003.1-igr3::*fdhSCL*, an experiment in shake flasks was performed using the RAMOS device and fructose concentrations between 80 g/L and 210 g/L. IK003.1-igr3::*fdhSCL* completely consumed fructose up to 210 g/L and the highest yield of 0.86 g/g was reached for the cultivation with 160 g/L fructose (Additional file 1: Fig. S5 and Table S2).

**Fig. 5:**
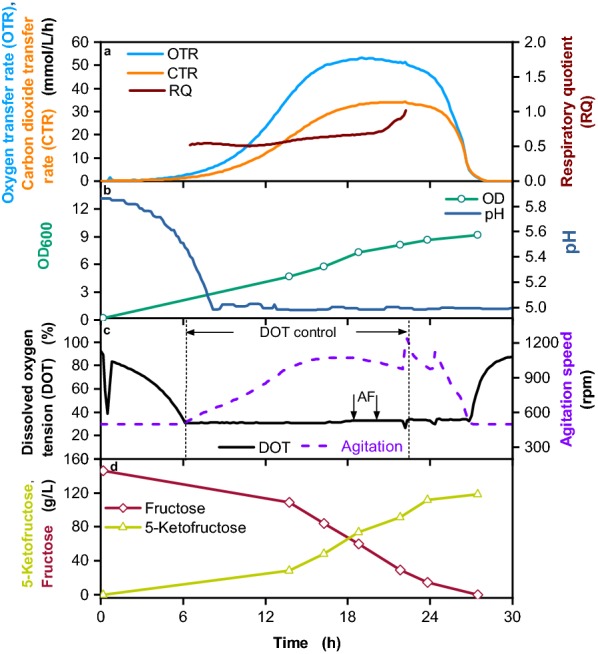
Cultivation of *G.* *oxydans* IK003.1-igr3::*fdhSCL* in a 2 L fermenter with pH control. Depicted is **a** the oxygen and carbon dioxide transfer rates (OTR and CTR), the respiratory quotient (RQ, shown for OTR values above 5 mmol/L/h), **b** optical density (OD_600_) and pH, **c** dissolved oxygen tension (DOT) and agitation speed, antifoam (AF) agent addition, and period of DOT control (indicated by arrows), **d** fructose and 5-KF concentrations. The cultivation was performed in complex medium with 150 g/L fructose with 1 L filling volume, DOT ≥ 30% controlled by agitation speed (500–1200 rpm), aeration rate (Q) =  L/min, T = 30 °C, pH_control _≥ 5

In a previous study, a 5-KF production process in a 2 L fermenter with the plasmid-based strain *G.* *oxydans* 621H pBBR1p264-*fdhSCL*-ST was described. This strain carried the same plasmid as IK003.1 pBBR1p264-*fdhSCL*-ST. *G. oxydans* 621H is not optimized for biomass formation from glucose and produces acetate rather than pyruvate. A batch fermentation with 150 g/L fructose was performed, reaching a 5-KF yield of 0.87 g/g [26]. To compare our genomic integration strain IK003.1-igr3::*fdhSCL* with this strain, a 2 L batch fermentation was performed under similar conditions (Fig. 5). An initial fructose concentration of 150 g/L was used to allow a direct comparison. Due to pH control by KOH addition, MES buffer was not included in the medium. The pH was set to 5, promoting both growth of *G.* *oxydans* and FDH activity. The DOT was maintained above 30% and antifoam was added twice during the cultivation. The OTR reached a maximum of 53 mmol/L/h after approx. 20 h and decreased sharply after 27 h, indicating the complete consumption of fructose (Fig. 5a). The OD_600_ increased over time and reached a final value of 9.2 (Fig. 5b), which is 1.7-fold higher compared with *G.* *oxydans* 621H pBBR1p264-*fdhSCL*-ST [26]. This difference is presumably caused by a somewhat higher fraction of fructose metabolized within the IK003.1 pBBR1p264-*fdhSCL*-ST cells and possibly also by the genetic alterations introduced into strain IK003.1 [17]. Most importantly, the 5-KF yield IK003.1-igr3::*fdhSCL* was 0.84 g/g and thus only slightly smaller than the 5-KF yield of 0.87 g/g determined for the plasmid-based strain [26].

### Shake flask cultivation of double integration strain IK003.1::*fdhSCL*^2^

**Fig. 6:**
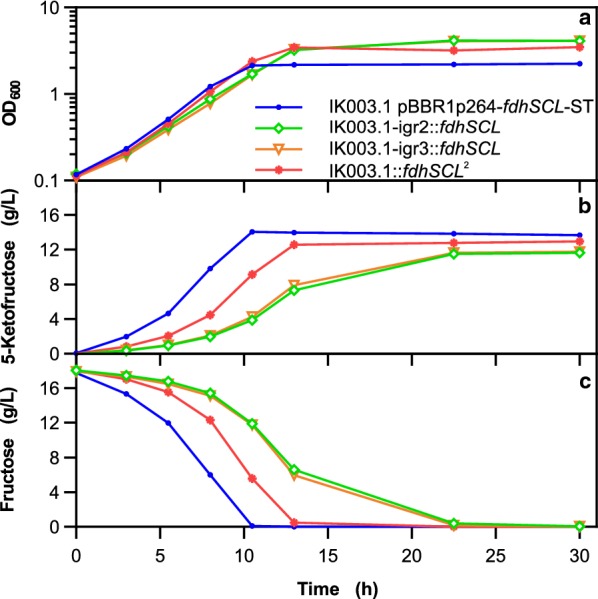
Growth, 5-KF formation, and fructose consumption of the indicated *G.* *oxydans* strains. Depicted are **a** the growth as OD_600_, **b** the 5-KF and **c** the fructose concentration (determined by HPLC method A). The strains were cultivated in 100 mL complex medium with 18 g/L fructose in 500 mL baffled shake flasks at 30 °C, 130 rpm, a shaking diameter of 50 mm and 85% humidity. Shown are mean values of biological duplicates

The results described above were obtained with integration strains containing a single genomic *fdhSCL* copy and a resulting lower expression rate compared to plasmid-based *fdhSCL* expression (see below). To further improve the potential for plasmid-free 5-KF production, the double integration strain IK003.1::*fdhSCL*^*2*^ was constructed using the two most potent integration sites igr3 and igr2 for *fdhSCL* integration. The performance of this strain was compared to the parent single integration strains IK003.1-igr2::*fdhSCL* and IK003.1-igr3::*fdhSCL* and to the plasmid-based strain IK003.1 pBBR1p264-*fdhSCL*-ST in a shake flask experiment. Growth curves, fructose consumption, and 5-KF formation are shown in Fig. 6.

The growth rate of strain IK003.1::*fdhSCL*^*2*^ (0.32 h^−1^) was close to that of the plasmid-based strain (0.33 h^−1^) and slightly higher than that of the single integration strains (0.28–0.29 h^−1^). The final OD_600_ after 30 h of the double integration strain (3.5) was lower than that of IK003.1-igr2::*fdhSCL* (4.1) and IK003.1-igr3::*fdhSCL* (4.1) and higher than that of the plasmid-based strain (2.3). Most importantly, the double integration strain IK003.1::*fdhSCL*^*2*^ was clearly faster than the single integration strains with respect to fructose consumption and 5-KF formation, but still slower than the plasmid-based strain. In a further experiment, the IK003.1::*fdhSCL*^*2*^ strain was compared with the parent single integration strains in a RAMOS cultivation device with 80 g/L fructose and 150 mM MES buffer (initial pH 6.0). As shown in Additional file 1: Fig. S6, the OTR kinetics confirmed faster fructose consumption by the double integration strain, reaching maximal OTR values at about 12 h, whereas the single integration strains reached the maximum after about 17 h. Under these conditions, IK003.1::*fdhSCL*^*2*^ reached a final 5-KF yield of 0.82 g/g, close to that of IK003.1 pBBR1p264-*fdhSCL* (0.84 g/g) obtained in the experiment shown in Fig. 4.

The properties of IK003.1::*fdhSCL*^*2*^ showed that doubling of the copy number of the *fdhSCL* genes and thus presumably a higher rate of *fdhSCL* transcription is sufficient to significantly increase the rate of fructose consumption and 5-KF formation. In the plasmid-based strain, the copy number of the *fdhSCL* genes is presumably above 20, as the copy number of the parent vector was determined to be 23 ± 6 in *G.* *oxydans* ∆*hsdR* [44]. In this case, the increase in *fdhSCL* copy number did not lead to tenfold higher rates of fructose consumption and 5-KF production, showing that there were other factors that limit fructose oxidation.

### Analysis of *fdhSCL* expression by RT-qPCR

**Fig. 7:**
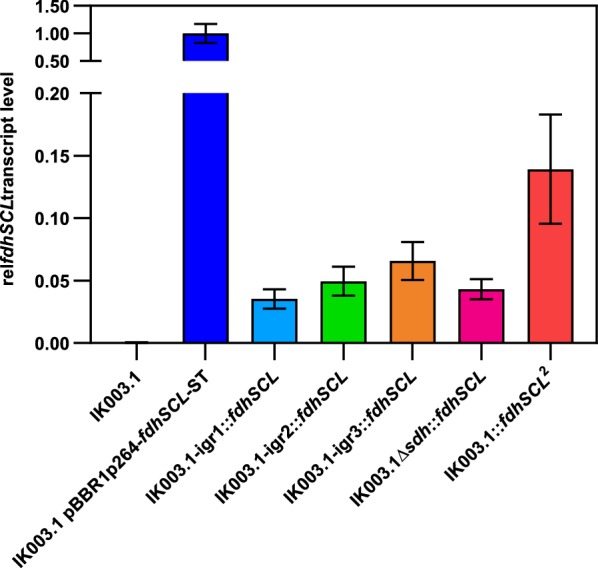
Relative *fdhSCL* transcript levels for the indicated *G.* *oxydans* strains as determined by RT-qPCR. Depicted is the transcript ratio *fdhSCL*/GOX0264 normalized to the transcript ratio of the plasmid-based strain IK003.1 pBBR1p264-*fdhSCL*-ST, which was set as 1.0. Shown are the mean values of at least three biological replicates and technical duplicates, with standard deviation as error bars

To validate that different *fdhSCL* transcription levels are responsible for the phenotypic differences of single integration strains, reverse transcription quantitative PCR (RT-qPCR) was performed with the single integration strains, the double integration strain, and the plasmid-based strain using RNA from cells in the exponential growth phase. The *gap* gene (GOX0508), encoding glyceraldehyde 3–phosphate dehydrogenase, was tested as reference. It has been used as reference gene for RT-qPCR in *G.* *oxydans* before [45, 46]. However, with this reference gene we failed to detect the difference between single integration strains and the double integration strain, suggesting that *gap* expression is not constant under the experimental conditions employed and thus not a suitable reference gene (data not shown). We therefore used the gene for a ribosomal protein as reference, namely GOX0264 encoding the ribosomal protein L35. The results obtained for the *fdhSCL*/GOX0264 mRNA ratios are shown in Fig. 7, normalized to the mRNA ratio of the plasmid-based strain (5.03), which was set as 1.

The *fdh* mRNA ratio of the four single integration strains varied between 3.5% and 6.6% of the value measured for the plasmid-based strain, which fits with a copy number of about 20 for pBBR1p264 in *G.* *oxydans* [44]. In the double integration strain, the *fdh* mRNA ratio was about twice as high (14% of the level of the plasmid-based strain), roughly corresponding to the sum of the values measured for IK003.1-igr2::*fdhSCL* and IK003.1-igr3::*fdhSCL* (12%). These results confirm the different expression levels expected from the *fdhSCL* copy numbers of the different strains. The *fdhSCL* transcription levels observed for the different integration strains correlated with the 5-KF production levels in the order IK003.1::*fdhSCL*^*2*^ > IK003.1-igr3::*fdhSCL* > IK003.1-igr2::*fdhSCL* > IK003.1 Δ*sdh*::*fdhSCL* > IK003.1-igr1::*fdhSCL*.

Despite the usage of the same promoter and terminator structures, *fdhSCL* expression in the four different integration sites varied, indicating that the genomic vicinity of the target gene had an influence on the expression. It has been reported that RNA polymerase activity increases with proximity to the origin of replication [47] and that transcription speed is influenced by the codon composition [48]. However, both factors are not applicable to the differences observed here for the four single integration strains, since all four sites are close to the origin and contain the same DNA sequence. Genome architecture and DNA supercoiling are known to influence bacterial gene expression with DNA gyrase playing an important role by introducing negative supercoils or relaxing positive supercoils introduced by RNA polymerase [47, 49, 50]. In *Escherichia* *coli,* an increase in gyrase cleavage sites was found downstream of highly transcribed operons [51]. Furthermore, nucleoid-associated proteins influence DNA folding and gene expression [52, 53]. Such mechanisms, which have not been studied at all in *G.* *oxydans*, are likely to contribute to the variation in *fdhSCL* expression at the four different integration sites.

## Conclusions

In this study, four different integration sites for heterologous genes were identified in the genome of *G.* *oxydans*, all enabling successful expression. The integration into the intergenic region of convergent genes flanked by terminators is thus a favorable option for future engineering studies requiring genomic expression of foreign genes. The position-dependent effects on gene expression observed in our experiments suggests that a comparison of different integration sites might be worthwhile. Chromosomal *fdhSCL* expression should enable stable expression and in fact we never observed loss of the ability for 5-KF production during growth for up to 30 generations and handling of the strains over several months. Furthermore, chromosomal expression avoids the necessity to use antibiotics required for plasmid-based *fdhSCL* expression. Plasmid loss can be an issue in the case of plasmid-based expression, however, no evidence for this has been observed in the case pBBR1p264-*fdhSCL*-ST. Another advantage of genomic *fdhSCL* expression is that it facilitates further metabolic engineering of the strains, for example toward utilization of alternative substrates. As doubling the genomic copy number of the *fdhSCL* genes correlated with twofold increased 5-KF synthesis rates, additional *fdhSCL* integrations could further increase 5-KF production. Also, the use of a promoter stronger than P264 could further enhance *fdhSCL* expression. Besides genetic optimization, process optimization is an obvious method to improve 5-KF production. In summary, our study provided the efficient *G.* *oxydans* strain IK003.1::*fdhSCL*^*2*^ for plasmid-free 5-KF production that will serve as basis for future strain and process development.

## Methods

### Strains, plasmids and oligonucleotides

All strains and plasmids used in this study are listed in Table 1. Oligonucleotides were obtained from Eurofins Genomics (Ebersberg, Germany) and are shown in Additional file 1: Table S3.

**Table 1:** Strains and plasmids used in this study

Strain or plasmid	Relevant characteristics	Source or references
*E. coli*		
DH5α	F– *endA1* Φ80d*lacZ*ΔM15 Δ(*lacZYA*-*argF*)U169 *recA1 relA1 hsdR17*(rK–mK +) *deoR supE44 thi*-*1 gyrA96 phoA* λ–, strain used for cloning	[57]
S17-1	Δ*recA*, *endA1*, *hsdR17*, *supE44*, *thi*-*1*, *tra *+ , strain used for conjugation of *G. oxydans*	[62]
*G. oxydans*		
IK003.1	*G. oxydans* 621H Δ*upp* Δ*gdhS*::*sdhCDABE* Δ*pdc*::*ndh* Δ*gdhM*::*sucCD*	[17]
IK003.1-igr1::*fdhSCL*	IK003.1 with the *fdhSCL* genes of *G. japonicus* under the control of the P264 promoter integrated between GOX0013 and GOX0014 (GOX_RS01200 and GOX_RS01205)	This work
IK003.1-igr2::*fdhSCL*	IK003.1 with the *fdhSCL* genes of of *G. japonicus* under the control of the P264 promoter integrated between GOX0028 and GOX0029 (GOX_RS01280 and GOX_RS01285)	This work
IK003.1-igr3::*fdhSCL*	IK003.1 with the *fdhSCL* genes of *G. japonicus* under the control of the P264 promoter integrated between GOX0038 and GOX0039 (GOX_RS01330 and GOX_RS01335)	This work
IK003.1 Δ*sdh*::*fdhSCL*	IK003.1 with the *fdhSCL* genes of *G. japonicus* under control of the P264 promoter replacing the genes GOX2095-6 (GOX_RS11750) encoding an inactive sorbitol dehydrogenase (authentic genomic frameshift)	This work
IK003.1::*fdhSCL*^*2*^	IK003.1-igr2::*fdhSCL* with an additional copy of the *fdhSCL* genes of *G. japonicus* under the control of the P264 promoter integrated between GOX0038 and GOX0039	This work
Plasmids		
pBBR1p264-*fdhSCL*-ST	pBBR1MCS-2 derivative containing the promoter region of GOX0264 upstream of the *fdhSCL* genes of *G. japonicus* NBRC3260 with a C-terminal Strep-tag II-encoding sequence fused to the 3′-end of *fdhL*	[30]
pAJ63a	Km^R^, FU^S^, derivative of pk18mobGII, integration vector for Δ*upp* based counter-selection	[35]
pAJ63a-igr1::*fdhSCL*	Km^R^, FU^S^, derivative of pAJ63a for integration of the *fdhSCL* genes under the control of the P264 promoter between GOX0013 and GOX0014 (GOX_RS01200 and GOX_RS01205)	This work
pAJ63a-igr2::*fdhSCL*	Km^R^, FU^S^, derivative of pAJ63a for integration of the *fdhSCL* genes under the control of the P264 promoter between GOX0028 and GOX0029 (GOX_RS01280 and GOX_RS01285)	This work
pAJ63a-igr3::*fdhSCL*	Km^R^, FU^S^, derivative of pAJ63a for integration of the *fdhSCL* genes under the control of the P264 promoter between GOX0038 and GOX0039 (GOX_RS01330 and GOX_RS01335)	This work
pAJ63a Δ*sdh*::*fdhSCL*	Km^R^, FU^S^, derivative of pAJ63a for integration of the *fdhSCL* genes under the control of the P264 promoter with simultaneous deletion of GOX2095-6 (GOX_RS11750)	This work

### Media composition

*G.* *oxydans* strains were cultivated in complex medium containing 5 g/L yeast extract (Karl Roth GmbH, Karlsruhe, Germany or BD Biosciences, Heidelberg, Germany), 2.5 g/L MgSO_4_ × 7 H_2_O, 1 g/L (NH_4_)_2_SO_4_, 1 g/L KH_2_PO_4_. The initial pH was adjusted to 6 with KOH [54]. The media were supplemented with 50 µg/mL cefoxitin and 10 µM thymidine, and for plasmid-carrying strains 50 µg/mL kanamycin was added. For the precultures, 40 g/L mannitol was added as carbon source and main cultures were conducted with different fructose concentrations as indicated in the respective experiments. *E.* *coli* strains were cultivated in lysogeny broth-based media [55] at 37 °C and 130 rpm, and 50 µg/mL kanamycin was added for plasmid-carrying strains.

### Generation of *fdhSCL* integration strains

To generate the *fdhSCL* integration strains, derivatives of pAJ63a [35] containing the *fdhSCL* genes of *G. japonicus* with promoter and terminator and flanked by about 500 bp DNA regions up- and downstream of the selected integration site were constructed. The required DNA fragments were amplified from suitable templates with the Phusion High Fidelity PCR Master Mix (New England Biolabs, Frankfurt am Main, Germany). The *fdhSCL* fragment with an ATG start codon for *fdhS* instead of the native TTG start codon was amplified from pBBR1p264-*fdhSCL*-ST [30] with the oligonucleotides RBS-ATG-fdhSCL-fwd, containing a consensus RBS, and fdhSCL-rev [33]. For all integration constructs, a 508 bp fragment covering the strong promoter of GOX0264 was amplified from pBBR1p264 [44] using the oligonucleotides P264-fwd and P264-rev-RBS-overlap with an overlap to the RBS and *fdhS*. A 100 bp bidirectional terminator region downstream of GOX0028 containing an overlap to *fdhL* was amplified with the oligonucleotides Term-GOX0028-fwd-fdhL-overlap and Term-GOX0028-rev from genomic DNA of strain IK003.1, which was isolated with the DNeasy Blood and Tissue Kit (Qiagen, Hilden, Germany). Two individual flanking regions of about 500 bp up- and downstream the respective integration sites were amplified from *G.* *oxydans* IK003.1 genomic DNA using specific oligonucleotide pairs with overlaps to the pAJ63a backbone, digested with PstI and KpnI, the promotor region and the terminator region to assemble all fragments via Gibson assembly [56]. The resulting fusion constructs were used to transform *E.* *coli* DH5α by the RbCl method [57]. Plasmids of positive clones were isolated using the QIAprep Spin Miniprep Kit (Qiagen, Hilden, Germany) and verified by sequencing (Eurofins Genomics, Ebersberg, Germany). Then the plasmids were transferred into the donor strain *E.* *coli* S17-1 for integration into *G.* *oxydans* IK003.1 via conjugation as described previously [17]. Positive clones, containing genomically integrated *fdhSCL* genes but not the vector backbone, were selected with 60 µg/mL 5-fluorouracile [35]. Positive clones were checked via colony PCR using a forward primer that binds upstream the upstream flanking region and a reverse primer, binding downstream of the downstream flanking region. Since the generation of the *fdhSCL* double integration strain turned out to be more challenging than the single integration strains, an adapted protocol was applied. IK003.1-igr2::*fdhSCL* was conjugated with *E.* *coli* S17–1 pAJ63a-igr3::*fdhSCL* according to the above mentioned protocol, while recombination media for the second recombination step and the agar plates for selecting the positive clones contained fructose instead of mannitol, as a strain with two chromosomal *fdhSCL* copies was assumed to show improved growth on fructose in comparison to a strain with a single *fdhSCL* copy. The double integration strain was obtained after several attempts.

### Cultivation in regular shake flasks

For biological replicates, individual 10 mL-precultures in 100 mL unbaffled shake flasks with complex medium containing 40 g/L mannitol were inoculated from agar plates and incubated for about 24 h at 30 °C and 250 rpm. Cells from these precultures were harvested for 15 min at 20 °C and 5.000*g*, washed once in the main culture medium and used to inoculate 100 mL main cultures in 500 mL baffled shake flasks to an OD_600_ (optical density at 600 nm) of 0.15 in complex medium with 18 g/L (100 mM) fructose. Main cultures were incubated at 30 °C and 130 rpm with a shaking diameter of 50 mm and 85% humidity (Shaker ISF1-X, Kuhner, Birsfelden, Switzerland).

### Cultivation in a respiration activity monitoring system (RAMOS)

Online monitoring of the respiratory activity in shake flask cultivations was performed using the Respiration Activity MOnitoring System (RAMOS) developed at the chair of biochemical engineering (RWTH Aachen University) [37, 38]. Commercial versions of the RAMOS device can be acquired from Kühner AG (Birsfeld, Switzerland) or HiTec Zang GmbH (Herzogenrath, Germany). Eight 250 mL shake flasks (unbaffled) were cultivated in parallel with an initial filling volume of 10 mL, 350 rpm shaking frequency and 50 mm shaking diameter (Climo-Shaker ISF1-X, Kuhner, Birsfelden, Switzerland). The aeration rate was set to 10 mL/min (1 vvm). Each flask is equipped with a partial pressure sensor for oxygen and a differential pressure sensor to determine oxygen and carbon dioxide transfer rates (OTR and CTR). The respiratory quotient (RQ) is the quotient of CTR and OTR. [37, 38]. For strain maintenance, glycerol stocks were used. Cells cultivated in complex medium with mannitol were harvested during the exponential growth phase, centrifuged and re-suspended in fresh preculture medium with 200 g/L glycerol. The glycerol stocks were stored at − 80 °C.

Precultures were inoculated with 100 µL glycerol stock suspension (OD_600_ = 2.4) and cultivated at 30 °C for 11 h to 19 h. Main cultures were inoculated from pre-cultures starting with an OD_600_ of 0.1. Preculture cells were centrifuged for 3 min at 16,214*g* and room temperature, and resuspended in main culture medium. Samples for offline analysis were taken from Erlenmeyer flasks operated at the same conditions in parallel to the online measurement. If necessary, 2-(*N*-morpholino) ethansulfonic acid (MES) buffer (pH 6, adjusted with 3 M KOH) was added in different concentrations to main cultures.

### Cultivation in 2 L bioreactors

Fermentation experiments were performed in a 2 L Sartorius BIOSTAT^®^ Bplus stirred tank reactor (Sartorius, Goettingen, Germany). The temperature was set to 30 °C and aeration was set to 1 L/min with a filling volume of 1 L (1 vvm). The fermenter was equipped with a six blade rusthton turbine and 4 baffles. During cultivation, the dissolved oxygen tension (DOT) was measured using a VisiFerm™ DO 225 pO_2_ sensor (Hamilton, Hoechst, Germany). The DOT was controlled at ≥ 30% by automatically adjusting the stirring rate between 500 rpm and 1500 rpm. The pH value was measured using a pH sensor (EasyFerm Plus K8 200, Hamilton, Hoechst, Germany). If necessary, pH was controlled with a 3 M KOH solution. Oxygen and carbon dioxide in the exhaust gas were measured using a DASGIP GA4 exhaust gas analyser (DASGIP, Eppendorf, Jülich, Germany). 0.5 mL antifoam agent Plurafac LF 1300 (BASF, Ludwigshafen, Germany) was added at the beginning of each experiment and when needed to prevent foaming. During fermentation, samples were taken from the bioreactor for offline analysis. Volume change by KOH titration and sampling were considered for mass balancing.

The performance of scale-up from shake flasks to the fermenter was investigated by cultivating simultaneously in both cultivation systems. For this purpose, media composition, pH, temperature and oxygen supply (aeration rate) were kept constant for both cultivation systems. The shake flasks were filled with medium from a fermenter. Cultivation broth was sterilely transferred from the fermenter into shake flasks to ensure that media composition, pH and optical density were identical [58–60].

### Offline analyses

The optical density of cultures at 600 nm (OD_600_) was measured either with a Genesys 20 photometer (Thermo Scientific, Darmstadt, Germany) or with an Ultraspec 2100 UV–Visible Spectrophotometer (biochrom, Holliston MA, USA). The photometers have a linear range between 0.1 and 0.3 and samples were diluted with 0.9% (w/v) NaCl if necessary. The pH values were determined with a HI221 Basic pH (Hanna Instruments Deutschland GmbH, Vöhringen, Germany), which was calibrated using two standard buffer solutions at pH 4 and 7. The osmolality was determined using the cryoscopic Osmometer OSMOMAT^®^ 030 (Genotec, Berlin, Germany).

Fructose consumption and 5-KF production were analyzed by HPLC of culture supernatants. 1 mL culture was centrifuged at 17,000*g* for 10 min and the supernatant was frozen at -20 °C until further analysis. Thawed samples were heated for 60 min at 60 °C (to avoid double peaks, probably caused by the existence of 5-ketofructose in an equilibrium of the keto and the germinal diol form [26]), filtered (0.2 µm syringe filter, Whatman™, GE Healthcare, Freiburg, Germany) and diluted with deionized water. Sugars were quantified using the same 5-KF standard with two different methods: HPLC method A: A 10 µL sample was measured in an Agilent LC-1100 system (Agilent, Santa Clara, CA, USA) equipped with a Carbo-Ca Guard Catridge (Phenomenex, Aschaffenburg, Germany) using a Rezex RCM-Monosaccharide 300, 7.8 mm column (Phenomenex, Aschaffenburg, Germany) for separation at 60 °C with water as eluent at a flow rate of 0.6 mL/min. 5-KF and fructose were detected with a refraction index detector (35 °C) [27]. 5-KF and fructose were calibrated in a range of 0.1 to 8 g/L, with retention times of 12.7 and 14.6 min, respectively. HPLC method B: An HPLC system of Shimadzu (Duisburg, Germany) was equipped with a precolumn Organic Acid Resin (40 × 8 mm, CS-Chromatographie Service, Langerwehe, Germany), the separating column Organic Acid Resin (250 × 8 mm, CS-Chromatographie Service, Langerwehe, Germany), and refraction index detector RID-20A (Shimadzu, Duisburg, Germany). The flow rate was set to 0.8 mL/min and an injection volume of 20 µL sample was used for analysis. 5 mM H_2_SO_4_ was used as mobile phase. Eluted components were detected and quantified using standard curves prepared with fructose and 5-KF solutions of known concentrations (0.032 g/L to 10 g/L). Evaporation during cultivations in the RAMOS device was determined gravimetrically and was taken into account for determination of fructose and 5-KF concentrations. Yields were calculated by dividing the produced 5-KF by the total fructose concentration and are indicated in g/g.

### Analysis of gene expression by RT-qPCR

Strains were cultivated in shake flasks as described above and harvested in the exponential growth phase (after 7.25 h). 35 mL culture were mixed with 15 g ice, centrifuged for 5 min at 5.000*g* and 4 °C and the resulting cell pellet was shock-frozen in liquid nitrogen and stored at − 80 °C. RNA was isolated using QIAzol Lysis Reagent (Qiagen, Hilden, Germany) including the following steps: cell disruption in a Precellys 24 homogenizer (Bertin, Frankfurt am Main, Germany), chloroform extraction, isopropanol precipitation, an on-column DNA digest on RNeasy mini spin columns, with RNase-free DNase (Qiagen, Hilden, Germany). Purified RNA was subjected to a second DNA digest using the Ambion™ DNAfree™ DNA Removal Kit (Thermo Scientific, Waltham, MA USA). RNA was checked for integrity after the first or second DNase digest to exclude strong RNA degradation using a High Sensitivity RNA ScreenTape in the TapeStation 2200 (Agilent, Santa Clara, CA USA). Final RNA concentrations were determined using the Colibri microvolume spectrometer (Titerek-Berthold, Pforzheim, Germany).

cDNA was generated with SuperScript™ III Reverse Transcriptase (Thermo Scientific, Braunschweig, Germany) with the following protocol. 10 ng RNA, 150 ng random primers (Invitrogen, Thermo Scientific, Braunschweig, Germany) and 1 µL of 10 mM dNTP mix (Invitrogen, Thermo Scientific, Braunschweig, Germany) in a total volume of 14 µL were incubated for 5 min at 65 °C and cooled for 1 min at 4 °C. Subsequently, 4 µL 5× first strand buffer, 1 µL 0.1 mM DTT, and 1 µL SuperScript III reverse transcriptase were added and incubated for 5 min at 25 °C, 60 min at 50 °C and finally for 5 min at 70 °C for inactivation of reverse transcriptase. For all samples, additionally a no amplification control was generated with water instead of reverse transcriptase to exclude genomic DNA contaminations in RT-qPCR. RT-qPCR was performed with a qTower instrument (Analytik Jena, Jena, Germany) using KAPA SYBR FAST qPCR Master Mix (Roche Diagnostics, Mannheim, Germany). 2 µL of cDNA or no template control were used for RT-qPCR. For each sample, at least three biological replicates with two technical replicates were measured.

To set up RT-qPCR, primers were designed using the online tool Primer3plus [61] and default qPCR settings with a target PCR product of 100–120 bp and an annealing temperature of 60 °C. Three different primer pairs for *fdhSCL*, two binding in *fdhS* and one in *fdhC*, one primer pair for GOX0264, and three primer pairs for *gap* (GOX0508) were designed and tested for PCR specificity. To optimize RT-qPCR conditions, all primer pairs were compared in a gradient from 57.1 to 63.9 °C regarding PCR specificity via agarose gel electrophoresis and melting curve analysis. The optimal temperature was determined to be 57.1 °C. Subsequently, the PCR efficiency was determined by running a RT-qPCR with an 8-step 1:10 dilution series of cDNA. Ct values were determined using qPCRsoft 3.1 (Analytik Jena). The optimal primer pairs were selected to quantitatively amplify cDNA fragments of *fdhC* (q-fdhC-fwd + q-fdhC-rev, PCR efficiency of 103.2%), of GOX0264 (q-GOX0264-fwd + q-GOX0264-rev, 103%), and of GOX0508 (q-gap-fwd2 + q-gap-rev2, 91.8%) Relative transcript levels were calculated from Ct values, considering the primer efficiencies, and normalized to the transcript levels of the plasmid strain.

## Supplementary information

**Additional file 1: Table S1.** Offline data for cultivation of the indicated *G. oxydans* strains with 80 g/L fructose in a RAMOS device shown in Fig. S1**. Table S2.** Offline date for the cultivation of *G. oxydans* IK003.1-igr3::*fdhSCL* in a RAMOS device with different fructose concentrations shown in Fig. S5. **Table S3.** Oligonucleotides used in this study. **Figure S1.** Cultivation of the indicated *G. oxydans* strains with 80 g/L fructose in a RAMOS device with online monitoring. Depicted are (**a**) the oxygen transfer rate (OTR) (**b**) the carbon dioxide transfer rate (CTR) (**c**) the respiratory quotient (RQ shown for OTR values above 5 mmol/L/h), (**d**) the total oxygen consumption (TOC) and (**e**) the total carbon dioxide evolution (TCE). The strains were cultivated in complex medium with 80 g/L fructose at 30 °C, 350 rpm, V_L_ = 10 mL in 250 mL flasks, pH_start_ = 6 and a shaking diameter of 50 mm. Shown are mean values of duplicates. **Figure S2.** Scale-up of batch fermentation of *G. oxydans* IK003.1-igr3::*fdhSCL* from shake flasks (RAMOS device) to a 2 L fermenter with 80 g/L fructose and 150 mM MES. Depicted are (**a**) oxygen transfer rate (OTR) and carbon dioxide transfer rate (CTR), (**b**) growth as OD_600_ and pH, (**c**) dissolved oxygen tension (DOT), agitation speed during fermentation, addition of antifoam agent (AF), and period of DOT control (indicated by arrows) and (**d**) fructose and 5-ketofructose concentration as determined by HPLC (method B). Cultivations were performed in complex medium with 80 g/L fructose prepared in the fermenter. The shake flask experiment was started with a sterile sample from the fermenter at 30 °C, 350 rpm, V_L_ = 10 mL in 250 mL flasks, pH_start_ = 6 and a shaking diameter of 50 mm using the RAMOS system. Fermentation was performed with 1 L filling volume in a L fermenter, DOT was kept ≥ 30% by variation of the agitation speed (500–1250 rpm), aeration rate (Q) = 1 L/min, 30 °C. **Figure S3.** Cultivation of *G. oxydans* IK003.1-igr3::*fdhSCL* in a 2 L fermenter with 100 mM MES and 80 g/L fructose. Depicted is (**a**) the oxygen and carbon dioxide transfer rates (OTR and CTR), (**b**) optical density (OD_600_) and pH, (**c**) dissolved oxygen tension (DOT) and agitation speed, and (**d**) fructose and 5-ketofructose concentrations. The cultivation was performed with 1 L filling volume, DOT ≥ 30% controlled by agitation speed (500–1500 rpm), aeration rate (Q) = 1 L/min, T = 30 °C. **Figure S4.** Cultivation of *G. oxydans* IK003.1-igr3::*fdhSCL* in a 2 L fermenter with 80 g/L fructose and pH control. Depicted is (**a**) the oxygen and carbon dioxide transfer rates (OTR and CTR), (**b**) optical density (OD_600_) and pH, (**c**) dissolved oxygen tension (DOT) and agitation speed, and (**d**) fructose and 5-ketofructose concentrations. The cultivation was performed with 1 L filling volume, DOT ≥ 30% controlled by agitation speed (500–1500 rpm), aeration rate (Q) = 1 L/min, T = 30 °C. **Figure S5.** Cultivation of *G. oxydans* IK003.1-igr3::*fdhSCL* in a RAMOS device with different fructose concentrations and 150 mM MES (initial pH of 6). Depicted is the oxygen transfer rate during growth with the indicated concentrations of fructose in complex medium at 30 °C, 350 rpm, V_L_ = 10 mL in 250 mL flasks, pH_start_ = 6 and a shaking diameter of 50 mm. Shown are mean values of duplicates. **Figure S6.** Cultivation of the indicated *G. oxydans* strains in a RAMOS device with 80 g/L fructose and 150 mM MES (initial pH of 6). Depicted is (**a**) the 5-ketofructose concentration (HPLC method B), the yield g/g, the OD_600_ and the final pH after 29 h and (**b**) the oxygen transfer rate (OTR). Cultivations were performed in complex medium with 80 g/L fructose and 150 mM MES buffer at 30 °C, 350 rpm, V_L_ = 10 mL in 250 mL flasks, pH_start_ = 6 and a shaking diameter of 50 mm. Shown are mean values of duplicates.

## Data Availability

The datasets supporting the conclusions of this article are included within the article and the additional file (Additional file1: Tables S1, S2, and S3, Figures S1, S2, S3, S4, S5, and S6).

## Abbreviations

5-KF: 5-Keto-d-fructose
CTR: Carbon dioxide transfer rate
DOT: Dissolved oxygen tension
DNA: Deoxyribonucleic acid
FAD: Flavin adenine dinucleotide
FDH: Fructose dehydrogenase
FPKM: Fragments Per Kilobase of transcript per Million mapped reads
HPLC: High-performance liquid chromatography
IGRs: Intergenic regions
MES: 2-(N-Morpholino)ethansulfonic acid
OD_600_: Optical density at 600 nm
ori: Origin of replication
OTR: Oxygen transfer rate
RAMOS: Respiration activity MOnitoring System
RBS: Ribosome binding site
RT-qPCR: Reverse transcription quantitative polymerase chain reaction
RQ: Respiratory quotient
TCA: Tricarboxylic acid cycle
TCE: Total carbon dioxide evolution
TOC: Total oxygen consumption
vvm: Vessel volume per minute
