# Supplementary material for: Novel plasmid-free Gluconobacter oxydans strains for production of the natural sweetener 5-ketofructose
Source: Microb Cell Fact. 2020 Mar 4;19:54. doi: 10.1186/s12934-020-01310-7 (PMC7055074; doi:10.1186/s12934-020-01310-7)
Supplement: Supplementary file 1 — Additional file 1: Table S1. Offline data for cultivation of the indicated G. oxydans strains with 80 g/L fructose in a RAMOS device shown in Fig. S1. Table S2. Offline date for the cultivation of G. oxydans IK003.1-igr3::fdhSCL in a RAMOS device with different fructose concentrations shown in Fig. S5. Table S3. Oligonucleotides used in this study. Figure S1. Cultivation of the indicated G. oxydans strains with 80 g/L fructose in a RAMOS device with online monitoring. Depicted are (a) the oxygen transfer rate (OTR) (b) the carbon dioxide transfer rate (CTR) (c) the respiratory quotient (RQ shown for OTR values above 5 mmol/L/h), (d) the total oxygen consumption (TOC) and (e) the total carbon dioxide evolution (TCE). The strains were cultivated in complex medium with 80 g/L fructose at 30 °C, 350 rpm, VL = 10 mL in 250 mL flasks, pHstart = 6 and a shaking diameter of 50 mm. Shown are mean values of duplicates. Figure S2. Scale-up of batch fermentation of G. oxydans IK003.1-igr3::fdhSCL from shake flasks (RAMOS device) to a 2 L fermenter with 80 g/L fructose and 150 mM MES. Depicted are (a) oxygen transfer rate (OTR) and carbon dioxide transfer rate (CTR), (b) growth as OD600 and pH, (c) dissolved oxygen tension (DOT), agitation speed during fermentation, addition of antifoam agent (AF), and period of DOT control (indicated by arrows) and (d) fructose and 5-ketofructose concentration as determined by HPLC (method B). Cultivations were performed in complex medium with 80 g/L fructose prepared in the fermenter. The shake flask experiment was started with a sterile sample from the fermenter at 30 °C, 350 rpm, VL = 10 mL in 250 mL flasks, pHstart = 6 and a shaking diameter of 50 mm using the RAMOS system. Fermentation was performed with 1 L filling volume in a L fermenter, DOT was kept ≥ 30% by variation of the agitation speed (500–1250 rpm), aeration rate (Q) = 1 L/min, 30 °C. Figure S3. Cultivation of G. oxydans IK003.1-igr3::fdhSCL in a 2 L fermenter with 100 mM MES an [file 12934_2020_1310_MOESM1_ESM.pdf]

Additional file 1

Novel plasmid-free *Gluconobacter oxydans* strains for production of the natural sweetener 5-ketofructose

Svenja Battling[1†], Karen Wohlers[2†], Chika Igwe[1], Angela Kranz[2], Matthias Pesch[1], Astrid Wirtz[2], Meike Baumgart[2], Jochen Büchs[1]* and Michael Bott[2]*

1 AVT-Biochemical Engineering, RWTH Aachen University,
Forckenbeckstraße 51, 52074 Aachen, Germany
2 IBG-1: Biotechnologie, Institut für Bio- und Geowissenschaften, Forschungszentrum Jülich GmbH, 52425 Jülich, Germany

*Correspondence: jochen.buechs@avt.rwth-aachen.de; m.bott@fz-juelich.de
†Svenja Battling and Karen Wohlers contributed equally to this manuscript

Table S1. Offline data for cultivation of the indicated *G. oxydans* strains with 80 g/L fructose in a RAMOS device

Table S2. Offline date for the cultivation of *G. oxydans* IK003.1-igr3::*fdhSCL* in a RAMOS device with different fructose concentrations

Table S3. Oligonucleotides used in this study.

Figure S1. Cultivation of the indicated *G. oxydans* strains with 80 g/L fructose in a RAMOS device with online monitoring.

Figure S2. Scale-up of batch fermentation of *G. oxydans* IK003.1-igr3::*fdhSCL* from shake flasks (RAMOS device) to a 2 L fermenter with 80 g/L fructose and 150 mM MES.

Figure S3. Cultivation of *G. oxydans* IK003.1-igr3::*fdhSCL* in a 2 L fermenter with 100 mM MES and 80 g/L fructose.

Figure S4. Cultivation of *G. oxydans* IK003.1-igr3::*fdhSCL* in a 2 L fermenter with 80 g/L fructose and pH control.

Figure S5. Cultivation of *G. oxydans* IK003.1-igr3::*fdhSCL* in a RAMOS device with different fructose concentrations and 150 mM MES (initial pH of 6)

Figure S6. Cultivation of the indicated *G. oxydans* strains in a RAMOS device with 80 g/L fructose and 150 mM MES (initial pH of 6).

Table S1. Offline data for cultivation of the indicated *G. oxydans* strains with 80 g/L fructose in a RAMOS device shown in Fig. S1.[1]

G. oxydans strain	Residual Fructose (g/L)	5-Ketofructose (g/L)	Yield (g/g)	OD_{600}	pH
IK003.1	69	3	0.04	3.6	3.8
IK003.1 pBBRp264-*fdhSCL*-ST	0	70	0.88	3.2	3.6
IK003.1-igr1::*fdhSCL*	45	29	0.36	3.2	3.4
IK003.1-igr2::*fdhSCL*	31	42	0.51	3.5	3.3
IK003.1-igr3::*fdhSCL*	25	47	0.58	3.8	3.3
IK003.1 Δ*sdh*::*fdhSCL*	32	38	0.47	3.6	3.3

[1] The data were measured after 29 h of cultivation. Fructose and 5-KF were determined by HPLC (method B). The yield represents $g_{5\text{-KF}}/g_{fructose,total}$. The strains were cultivated in complex medium with 80 g/L fructose at 30 °C, 350 rpm and a shaking diameter of 50 mm.

Table S2. Offline date for the cultivation of *G. oxydans* IK003.1-igr3::*fdhSCL* in a RAMOS device with different fructose concentrations shown in Fig. S5.[1]

Fructose (g/L)	Osmolality (Osmol/kg)	5-Ketofructose (g/L)	Yield (g/g)	OD_{600}	pH
80	0.8	60	0.77	5.30	5.0
100	0.9	75	0.77	5.30	4.9
120	1.1	96	0.80	5.00	4.8
160	1.4	141	0.86	4.60	4.7
180	1.6	150	0.84	4.00	4.7
210	1.8	164	0.78	3.50	4.9

[1]The values for 5-KF, yield, OD_{600} and pH were measured after 25 h (80 g/L -120 g/L fructose) and after 72 h (160 g/L – 210 g/L fructose).

Table S3. Oligonucleotides used in this study

Oligonucleotide	Sequence (5'→ 3') and properties[a]
**Oligonucleotides for Cloning**	
P264-fwd	CGTTGCGCCTGAATGAGAGGAAAG
P264-rev-RBS-overlap	**CCATCTGCAGTCCTCCTTTC**TTCGGTCTCCCTCGCCGTAAAC
RBS-ATG-fdhSCL-fwd	**GAAAGGAGGACTGCAGA**TGGAAAAAATAGCTGATTCCGGCCCTG
fdhSCL-rev	TTACCCCTGTTTCAGGTCATTGAG
Term-GOX0028-fwd-fdhL-overlap	**ATGACCTGAAACAGGGGTAA**GCGCGTTCCCGAGCGGTTC
Term-GOX0028-rev	AGACTGAAGTCTCGGTTCAGACAGATAAAAAAAG
FlA-igr1-fwd-pAJ-overlap	**TACGAATTCGAGCTCGGTAC**AACCTGACCAGCTCAACACTGGG
FlA-igr1-rev-fdhSCL-overlap	**CCTCTCATTCAGGCGCAACG**CCTCGTGAACTGAAAGACCG
FlB-igr1-fwd-Term-overlap	**CTGAACCGAGACTTCAGTCT**ATATCTCGCTCCTGCCCTGTG
FlB-igr1-rev-pAJ63a-overlap	**GCCAAGCTTGCATGCCTGCA**TTACGCTTATGCGTTCGCGCC
FlA-igr2-fwd-pAJ-overlap	**TACGAATTCGAGCTCGGTAC**TCTCACTTCAGCGCCGCCATC
FlA-igr2-rev-fdhSCL-overlap	**CCTCTCATTCAGGCGCAACG**AGACAGATAAAAAAAGCCGGTCCCGC
FlB-igr2-fwd-Term-overlap	**CTGAACCGAGACTTCAGTCT**GAACCGAGACTTCAGTCTGC
FlB-igr2-rev-pAJ63a-overlap	**GCCAAGCTTGCATGCCTGCA**CACGTCATCATGAAAGTGCATC
FlB-igr3-fwd-Term-overlap	**TACGAATTCGAGCTCGGTAC**CATCTGGCCGCCCATCCC
FlB-igr3-rev-pAJ63a-overlap	**CCTCTCATTCAGGCGCAACG**GCACTAATCCGAAAAGAGCGGTTG
FlB-igr3-fwd-Term-overlap	**CTGAACCGAGACTTCAGTCT**TCGATCAGACCTGTGTGTTC
FlB-igr3-rev-pAJ63a-overlap	**GCCAAGCTTGCATGCCTGCA**TTACGACATGGAACCGGGC
FlB-sdh-fwd-Term-overlap	**TACGAATTCGAGCTCGGTAC**CAGAGCCTGCAACCGGCG
FlB-sdh-rev-pAJ63a-overlap	**CCTCTCATTCAGGCGCAACG**TTTGATCTGAAGACATAGGAGATGCTG
FlB-sdh-fwd-Term-overlap	**CTGAACCGAGACTTCAGTCT**TGGTCTTTCCTTATTGGTGGAACGG
FlB-sdh-rev-pAJ63a-overlap	**GCCAAGCTTGCATGCCTGCA**TCGCCGGTTTCCTGCTTCTC
**Oligonucleotides for sequencing and colony-PCR**	
pAJ63a-seq-fwd	TGCTTCCGGCTCGTATGTTG
pAJ63a-seq-rev	GGATGTGCTGCAAGGCGATTAAG
fdh-seq-fwd2	GTGGTTATGCCATTTCTTCCCC
fdh-seq-fwd3	AACCGATGGTGCTGCACTC
fdh-seq-fwd4	CCCGCATACCAACTACTTCC
fdh-seq-rev	CCGGCATGACATCGACGG
Integr-igr1-seq-fwd	TGGCAATATTCTCGGCTTCAC
Integr-igr1-seq-rev	CGTGATCGAAACGCCTCTGC
Integr-igr2-seq-fwd	TCACCGCCACAGGCTTTG
Integr-igr2-seq-rev	GCTGGGTTCAGCCATAGC
Integr-igr3-seq-fwd	CAGCGAGGCCTATGCCAAAC
Integr-igr3-seq-rev	GATGATGCGGGCCTGGAC
Integr-sdh-seq-rev	GCTGATGCGGATGTCACGTC
Integr-sdh-seq-fwd	CGGGGTGTGTGGCATGTC
**Oligonucleotides for RT-qPCR**	
q-fdhS-fwd1	AGCCTAACAGTCGCAGCAAT
q-fdhS-rev1	AAGCGGAAAGCTGCATAAAA
q-fdhS-fwd2	CCCACGCTCATCCAGATTAT
q-fdhS-rev2	GGGACGGCTGATACATGAGT
q-fdhC-fwd	TACCCACAATGACGACTGGA
q-fdhC-rev	ACTGATTGGGCGCTAGAAGA
q-GOX0264-fwd	AGACCAAGTCGTCGGTCAAG
q-GOX0264-rev	GTTGGTGCGCTTCATCTTCT
q-gap-fwd1	TCCGACTTCAACCATGACAA
q-gap-rev1	TTGTCGTACCACGAGCAGAC
q-gap-rev1b	GTTGTCGTACCACGAGCAGA
q-gap-fwd2	ATGATCAGGCTGTTGCCTTC
q-gap-rev2	TTGTCGCCATCAACGATCTG

[a] overlaps for Gibson Assembly in bold

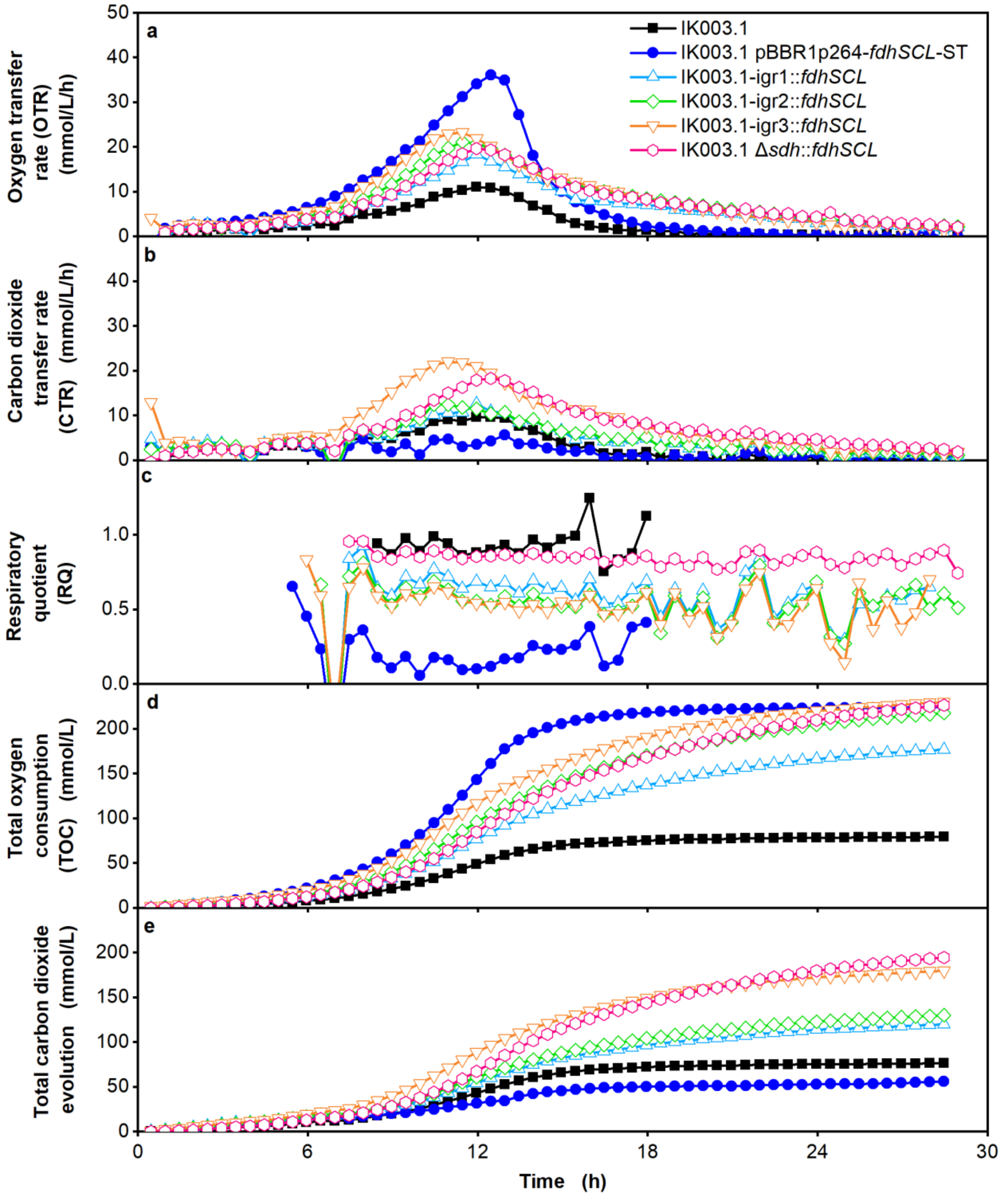

Figure S1. Cultivation of the indicated *G. oxydans* strains with 80 g/L fructose in a RAMOS device with online monitoring. Depicted are (**a**) the oxygen transfer rate (OTR), (**b**) the carbon dioxide transfer rate (CTR), (**c**) the respiratory quotient (RQ shown for OTR values above 5 mmol/L/h), (**d**) the total oxygen consumption (TOC) and (e) the total carbon dioxide evolution (TCE). The strains were cultivated in complex medium with 80 g/L fructose at 30 °C, 350 rpm, V_L = 10 mL in 250 mL flasks, pH_{start} = 6 and a shaking diameter of 50 mm. Shown are mean values of duplicates.

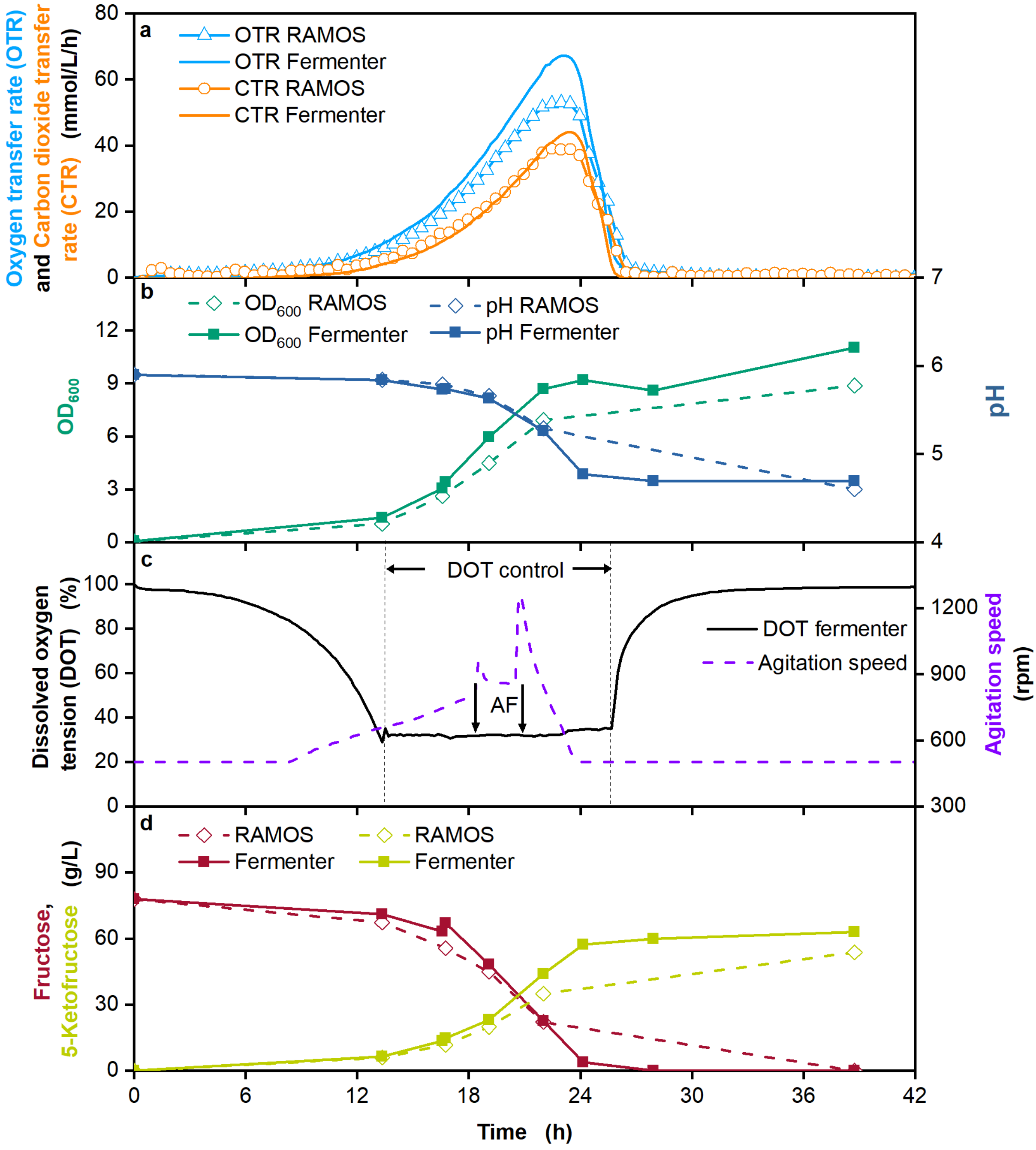

Figure S2. Scale-up of batch fermentation of *G. oxydans* IK003.1-igr3::*fdhSCL* from shake flasks (RAMOS device) to a 2 L fermenter with 80 g/L fructose and 150 mM MES. Depicted are (**a**) oxygen transfer rate (OTR) and carbon dioxide transfer rate (CTR), (**b**) growth as OD_{600} and pH, (**c**) dissolved oxygen tension (DOT), agitation speed during fermentation, addition of antifoam agent (AF), and period of DOT control (indicated by arrows) and (**d**) fructose and 5-ketofructose concentration as determined by HPLC (method B). Cultivations were performed in complex medium with 80 g/L fructose prepared in the fermenter. The shake flask experiment was started with a sterile sample from the fermenter at 30 °C, 350 rpm, V_L = 10 mL in 250 mL flasks, pH_{start} = 6 and a shaking diameter of 50 mm using the RAMOS system. Fermentation was performed with 1 L filling volume in a 2 L fermenter, DOT was kept ≥ 30 % by variation of the agitation speed (500 rpm – 1250 rpm), aeration rate (Q) = 1 L/min, 30 °C.

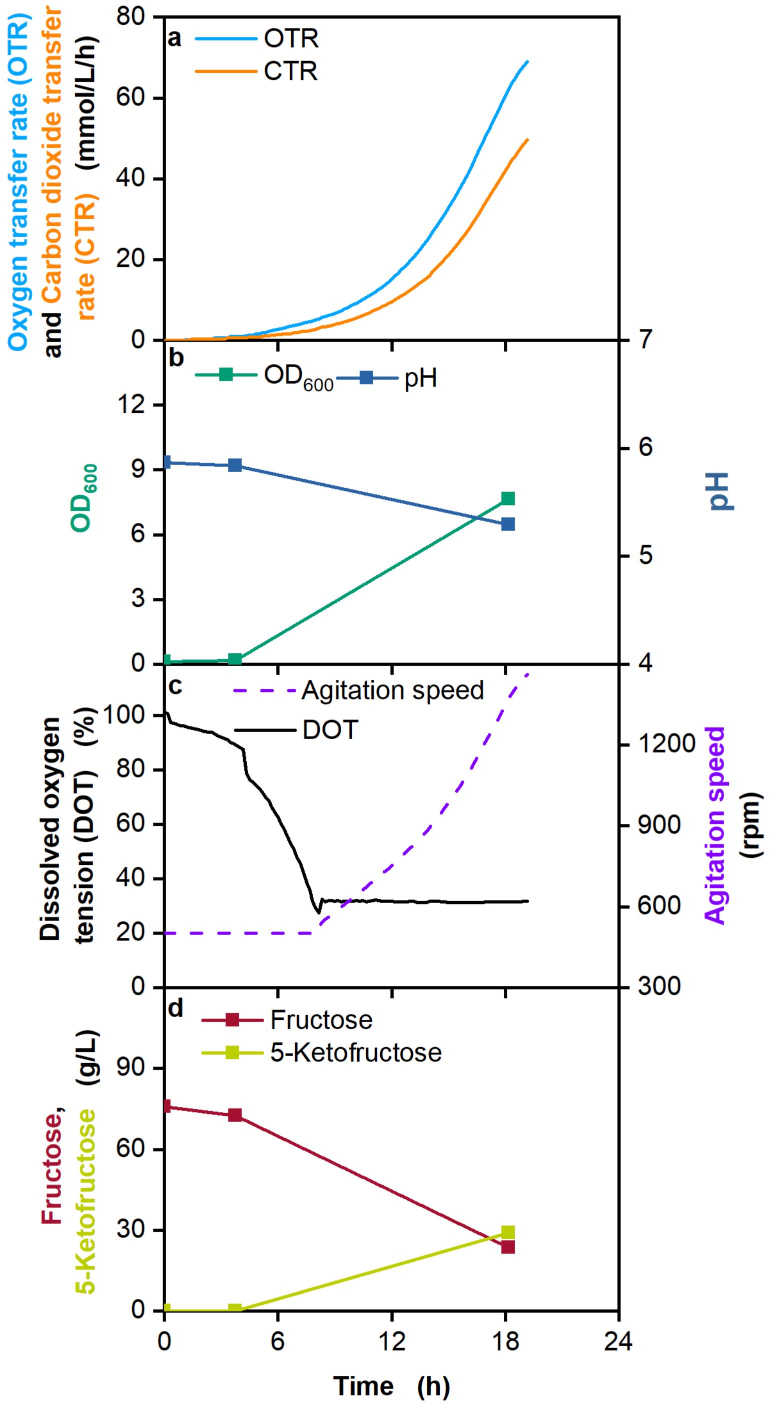

Figure S3. Cultivation of *G. oxydans* IK003.1-igr3::*fdhSCL* in a 2 L fermenter with 100 mM MES and 80 g/L fructose. Depicted is (**a**) the oxygen and carbon dioxide transfer rates (OTR and CTR), (**b**) optical density (OD_{600}) and pH, (**c**) dissolved oxygen tension (DOT) and agitation speed, (**d**) fructose and 5-ketofructose concentrations. The cultivation was performed with 1 L filling volume, DOT ≥ 30 % controlled by agitation speed (500 rpm – 1500 rpm), aeration rate (Q) = 1 L/min, T = 30 °C.

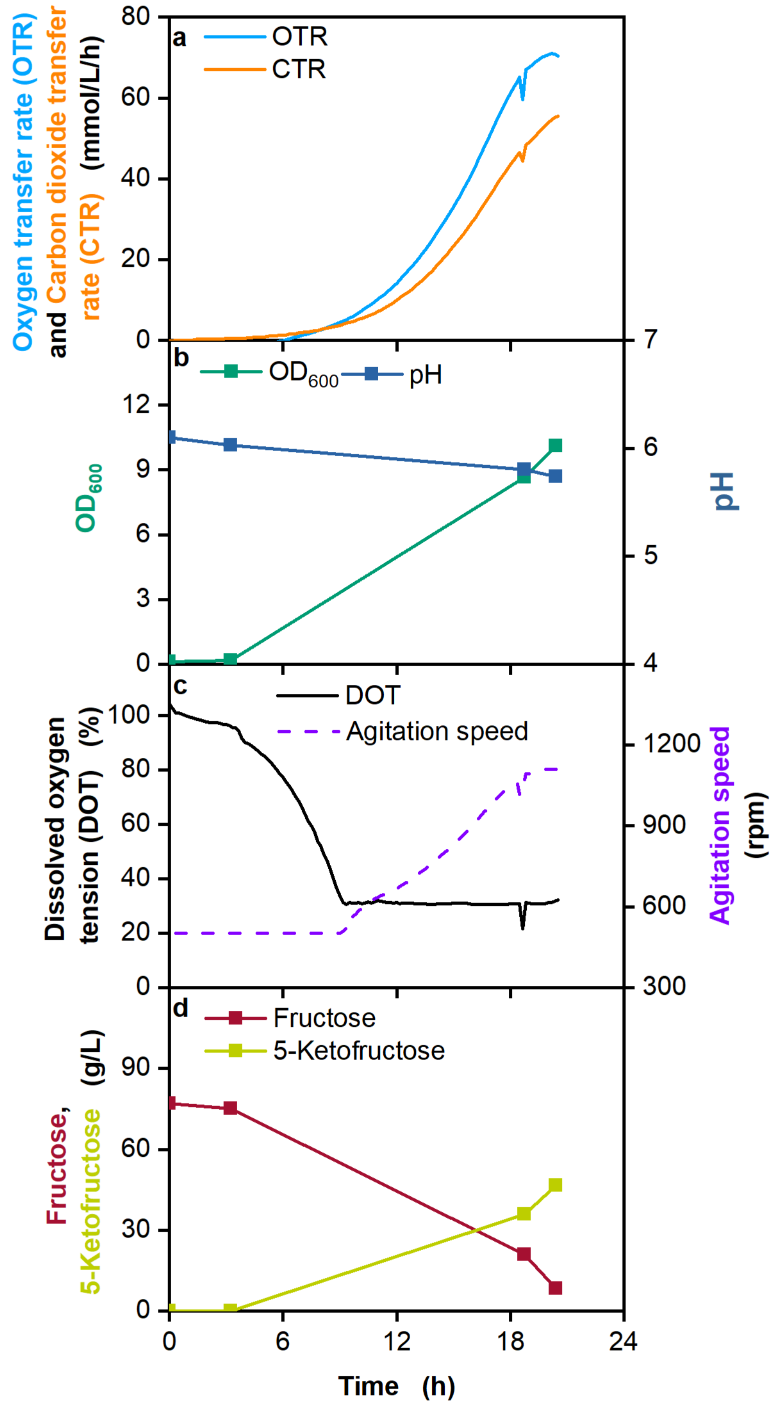

Figure S4. Cultivation of *G. oxydans* IK003.1-igr3::*fdhSCL* in a 2 L fermenter with 80 g/L fructose and pH control. Depicted is (**a**) the oxygen and carbon dioxide transfer rates (OTR and CTR), (**b**) optical density (OD_{600}) and pH, (**c**) dissolved oxygen tension (DOT) and agitation speed, (**d**) fructose and 5-ketofructose concentrations. The cultivation was performed with 1 L filling volume, DOT ≥ 30 % controlled by agitation speed (500 rpm – 1500 rpm), aeration rate (Q) = 1 L/min, T = 30 °C.

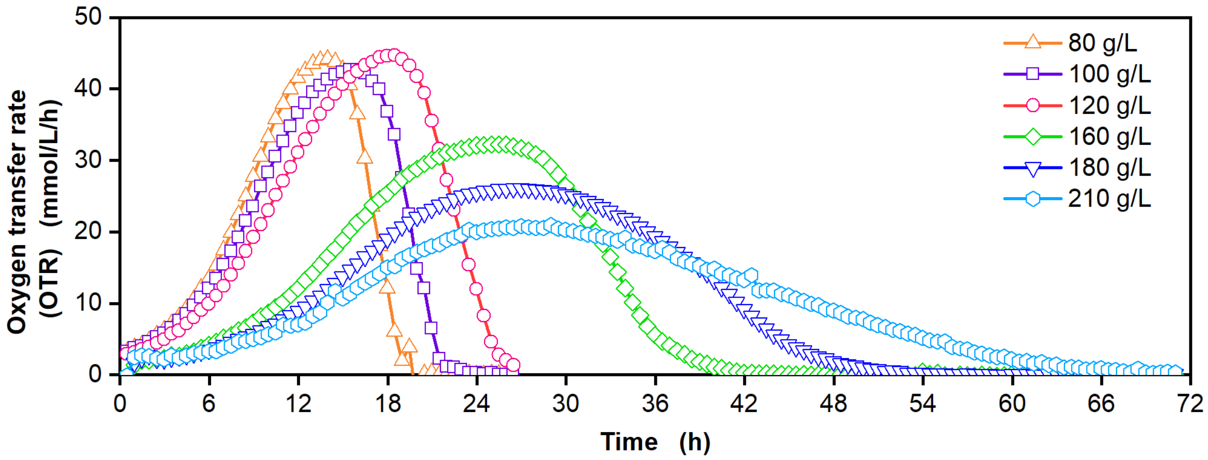

Figure S5. Cultivation of *G. oxydans* IK003.1-igr3::*fdhSCL* in a RAMOS device with different fructose concentrations and 150 mM MES (initial pH of 6). Depicted is the oxygen transfer rate during growth with the indicated concentrations of fructose in complex medium at 30 °C, 350 rpm, V_L = 10 mL in 250 mL flasks, pH_{start} = 6 and a shaking diameter of 50 mm. Shown are mean values of duplicates.

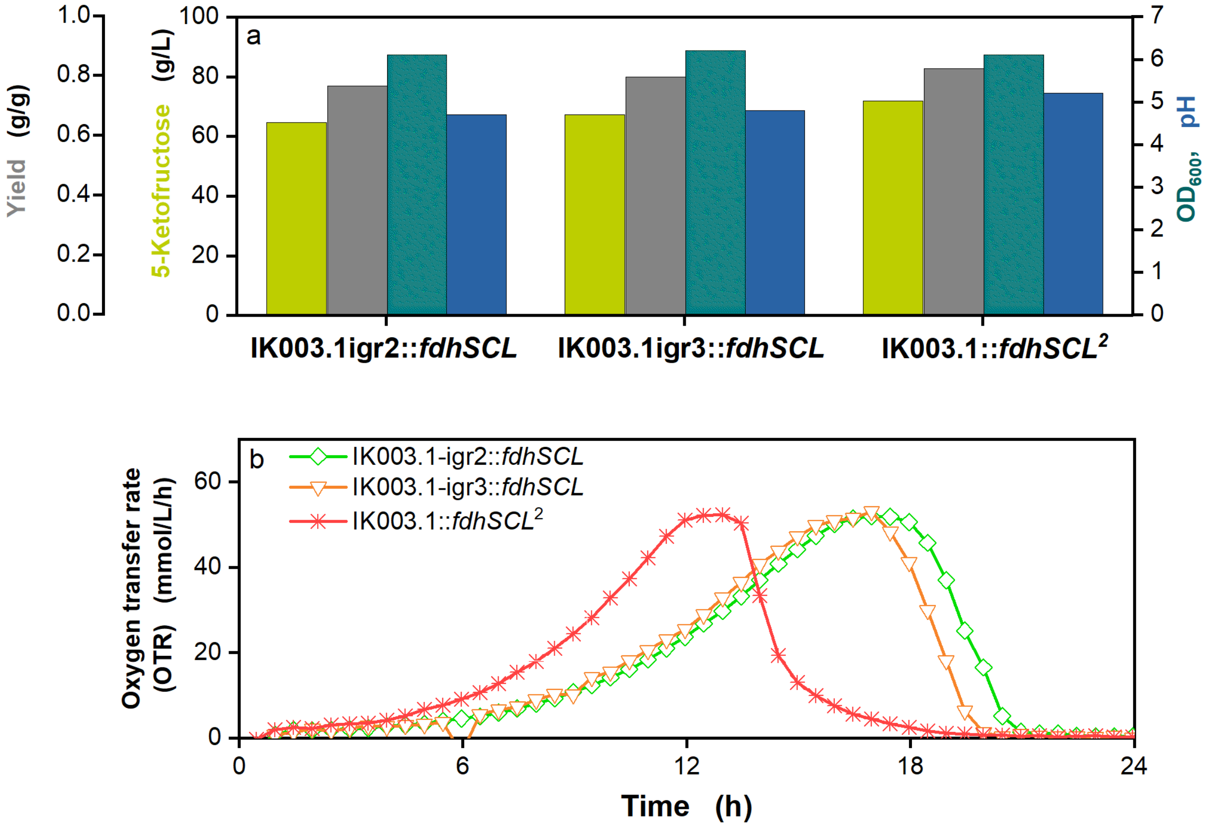

Figure S6. Cultivation of the indicated *G. oxydans* strains in a RAMOS device with 80 g/L fructose and 150 mM MES (initial pH of 6). Depicted is (**a**) the 5-ketofructose concentration (HPLC method B), the yield g/g$_{l}$, the OD_{600} and the final pH after 29 h and (**b**) the oxygen transfer rate (OTR). Cultivations were performed in complex medium with 80 g/L fructose and 150 mM MES buffer at 30 °C, 350 rpm, V_L = 10 mL in 250 mL flasks, pH_{start} = 6 and a shaking diameter of 50 mm. Shown are mean values of duplicates.
